# Age-Related Cognitive and Motor Decline in a Mouse Model of CDKL5 Deficiency Disorder is Associated with Increased Neuronal Senescence and Death

**DOI:** 10.14336/AD.2020.0827

**Published:** 2021-06-01

**Authors:** Laura Gennaccaro, Claudia Fuchs, Manuela Loi, Riccardo Pizzo, Sara Alvente, Chiara Berteotti, Leonardo Lupori, Giulia Sagona, Giuseppe Galvani, Antonia Gurgone, Alessandra Raspanti, Giorgio Medici, Marianna Tassinari, Stefania Trazzi, Elisa Ren, Roberto Rimondini, Tommaso Pizzorusso, Giovanna Zoccoli, Maurizio Giustetto, Elisabetta Ciani

**Affiliations:** ^1^Department of Biomedical and Neuromotor Sciences, University of Bologna, Bologna, Italy.; ^2^Department of Neuroscience, University of Turin, Turin, Italy.; ^3^BIO@SNS lab, Scuola Normale Superiore di Pisa, Pisa, Italy.; ^4^Institute of Neuroscience, National Research Council, Pisa, Italy.; ^5^Department of Neuroscience, Psychology, Drug Research and Child Health NEUROFARBA, University of Florence, Florence, Italy.; ^6^Department of Developmental Neuroscience, IRCCS Stella Maris Foundation, Pisa, Italy.; ^7^Department of Medical and Clinical Sciences, University of Bologna, Bologna, Italy.; ^8^National Institute of Neuroscience-Italy, Turin, Italy.

**Keywords:** CDKL5, neuronal death, neuronal senescence, DNA damage, γH2AX, XRCC5

## Abstract

CDKL5 deficiency disorder (CDD) is a severe neurodevelopmental disease caused by mutations in the X-linked *CDKL5* gene. Children affected by CDD display a clinical phenotype characterized by early-onset epilepsy, intellectual disability, motor impairment, and autistic-like features. Although the clinical aspects associated with *CDKL5* mutations are well described in children, adults with CDD are still under-characterized. Similarly, most animal research has been carried out on young adult *Cdkl5* knockout (KO) mice only. Since age represents a risk factor for the worsening of symptoms in many neurodevelopmental disorders, understanding age differences in the development of behavioral deficits is crucial in order to optimize the impact of therapeutic interventions. Here, we compared young adult *Cdkl5* KO mice with middle-aged *Cdkl5* KO mice, at a behavioral, neuroanatomical, and molecular level. We found an age-dependent decline in motor, cognitive, and social behaviors in *Cdkl5* KO mice, as well as in breathing and sleep patterns. The behavioral decline in older *Cdkl5* KO mice was not associated with a worsening of neuroanatomical alterations, such as decreased dendritic arborization or spine density, but was paralleled by decreased neuronal survival in different brain regions such as the hippocampus, cortex, and basal ganglia. Interestingly, we found increased β-galactosidase activity and DNA repair protein levels, γH2AX and XRCC5, in the brains of older *Cdkl5* KO mice, which suggests that an absence of Cdkl5 accelerates neuronal senescence/death by triggering irreparable DNA damage. In summary, this work provides evidence that CDKL5 may play a fundamental role in neuronal survival during brain aging and suggests a possible worsening with age of the clinical picture in CDD patients.

CDKL5 deficiency disorder (CDD, OMIM #300203) is a rare X-linked neurodevelopmental disorder caused by mutations in the cyclin-dependent kinase-like 5 gene (*CDKL5*). Although the clinical manifestations of individuals with the *CDKL5* mutation closely resemble some features of Rett syndrome (RTT) [1], including hand stereotypies, autistic-like features, scarce acquisition of language and conscious hand use, it has now become clear that CDD represents an independent clinical entity with unique characteristics [2, 3]. The clinical features commonly associated with *CDKL5* mutations include early-onset seizures, cortical visual impairment, intellectual disability, and gross motor impairment [4-8]. Even if the clinical aspects associated with *CDKL5* mutations are well described in children, adults with CDD are still under-characterized.

CDKL5 is highly expressed in the brain, in particular in the forebrain regions such as the cerebral cortex and the hippocampus; the expression of this gene is mainly neuronal, suggesting its potential importance in brain function. To delineate how CDKL5 deficiency leads to impaired brain function, several cellular and animal models of the disease have been created [9-12]. Mouse models of CDD (*Cdkl5* knockout (KO) mice) recapitulate multiple clinical symptoms of the disorder, such as motor dysfunction, cognitive deficits, visual impairment, and altered anxiety-related behaviors. Studies of brain development and function in CDD mouse models have demonstrated that CDKL5 plays a key role in neuronal morphogenesis, dendritic arborization of hippocampal and cortical principal neurons, and in synaptic connectivity [9, 13-18]. CDKL5 deficiency reduces spine maturation, a defect that is consistent with a reduced expression of the AMPA-R GluA2 subunit [16, 19, 20]. In addition, long-term plasticity of excitatory synapses is impaired in cortical slices of *Cdkl5* KO mice, and excitatory synaptic transmission is reduced in mouse hippocampal cultures silenced for *Cdkl5* [21]. This evidence strongly suggests that CDKL5 plays a central role in neuronal morphogenesis and excitatory synaptic transmission and implies that defective neuronal circuit function underlies CDD.

Recent evidence has highlighted the role of CDKL5 in neuronal survival [14, 22, 23]. It has been demonstrated that in the postnatal developing mouse hippocampus, loss of Cdkl5 increases the proliferation rate of neuronal precursors but causes the death of immature newborn neurons, thereby modulating the intricate balance between precursor proliferation and survival. Interestingly, CDKL5 deficiency increases the vulnerability of neural cells to different neurotoxic/ excitotoxic stimuli, suggesting that CDKL5 mutations have an endangering action that is likely to sensitize neurons in the brain to neurotoxic conditions known to promote neuronal death.

Although age is a risk factor for the worsening of symptoms in many neurodevelopmental disorders, such as Down syndrome [24], Fragile X syndrome [25, 26], and autism spectrum disorders [27, 28], the age-related phenotype in CDD has not yet been investigated. In the current study, we address the question of whether there are changes to the behavioral and neuroanatomical phenotypes in CDD as a function of age, using a well-characterized mouse model of CDD, the *Cdkl5* KO mouse [9].

## MATERIALS AND METHODS

### Colony

The mice used in this work derive from the *Cdkl5* KO strain in the C57BL/6N background developed in [9] and backcrossed in C57BL/6J for three generations. *Cdkl5* KO mice (-/Y) and age matched controls (wild-type; +/Y), when possible littermates, were used for all experiments and genotyped as previously described [9]. Mice were housed three to five animals per cage and maintained on a 12 h light: 12 h dark cycle in a temperature- (23°C) and humidity-controlled environment with standard mouse chow and water *ad libitum*. The animals’ health and comfort were controlled by the veterinary service. All research and animal care procedures were performed according to protocols approved by the Italian and European Community law for the use of experimental animals and by Bologna University Bioethical Committee. In this study, all efforts were made to minimize animal suffering and to keep the number of animals used to a minimum. A total of 147 animals were used for behavioral, neuroanatomical, and molecular studies. The number of mice included in individual analyses is reported in Supplementary Table 1.

### Behavioral Assays

The sequence of the tests was arranged to minimize the effect of one test influencing subsequent evaluation of the next test, and mice were allowed to recover for several days between different tests. All behavioral studies and analyses were performed blind to genotype. Mice were allowed to habituate to the testing room for at least 1 h before the test, and testing was always performed at the same time of the day.

*Marble Burying* - The marble burying test was performed by placing animals individually in a cage (dimensions 45?x 23?cm) with 5 cm of unscented standard bedding material. Before testing, animals were allowed to habituate to the novel environment for 5 min. After mice were gently removed from the cage and twenty marbles (15 mm in diameter) were spaced out equally on the top of the bedding in a matrix of five rows per four columns. Animals were allowed to explore the cage with marbles undisturbed for 30?min and at the end of the test, animals were gently removed from the cage to avoid disturbing the bedding. The number of marbles that were at least two-thirds buried were counted.

**Table1:** Body weight (mean ± SD) in grams of wild type (*Cdkl5* +/Y) and knockout (*Cdkl5* -/Y) male mice at different ages.

Months	*Cdkl5* +/Y	*Cdkl5* -/Y	p
1	14.82 ± 0.71 (n = 12)	14.94 ± 0.36 (n = 12)	n.s.
2	21.29 ± 0,49^*^ (n = 12)	21.02 ± 0.41^*^ (n = 12)	n.s.
3-4	25.53 ± 0.55^**^ (n = 12)	25.41 ± 0.37^**^ (n = 12)	n.s.
6-7	27.76 ± 0.77^***^ (n = 12)	28.60 ± 0.59^***^ (n = 12)	n.s.
12-14	31.81 ± 0.53^****^ (n = 12)	33.04 ± 0.62^****^ (n = 12)	n.s.

* Significantly different from 1 month;

** significantly different from 2 months;

*** significantly different from 3-4 months;

**** significantly different from 6-7 months. n.s. not statistically significant. Difference considered significant when p < 0.05 (Duncan’s test after ANOVA).

*Hind-limb Clasping -* Animals were suspended by their tail for 2 min and hind-limb clasping was assessed independently by two operators from video recordings. A clasping event is defined as the retraction of limbs into the body and toward the midline. The time spent hind-limb clasping was expressed as a percentage.

*Accelerating Rotarod Assay. -* Before the first test session, animals were briefly (30 s) trained at a constant speed of 5 rpm on the rotarod apparatus (Ugo Basile). Testing was performed at an accelerating linear speed (5-35 rpm within 270 s + 30 s max speed). Four testing trials with an intertrial interval of 1 h were performed. The number of passive rotations (rotation in which the mouse does not perform any coordinated movement but is passively transported by the rotating apparatus) were recorded for each trial. The mean frequency of passive rotations in the four testing trials was calculated and expressed as fold of the wild-type counterpart of the same age.

*Morris Water Maze -* Mice were trained to locate a hidden escape platform in a circular pool. The apparatus consisted of a circular water tank (1 m in diameter, 50 cm high) with a transparent round escape platform (10 cm^2^) placed in a fixed position. The tank was filled with tap water at a temperature of 22°C up to 0.5 cm above the top of the platform, and the water was made opaque with milk. In the experimental room, intra-maze and extra-maze visual cues were placed to enable spatial orientation. Mouse behavior was automatically video-tracked (EthoVision 3.1; Noldus Information Technology B.V.). During training, each mouse was subjected to either 1 swimming session of 4 trials (day 1) or 2 sessions of 4 trials per day (days 2-5), with an intersession interval of 1 h (acquisition phase). Mice were allowed to search for the platform for up to 60 s. If a mouse did not find the platform, it was gently guided to it and allowed to remain there for 15 s. During the intertrial interval (15 s), mice were placed in an empty cage. The latency to find the hidden platform was used as a measure of learning. Twenty-four h after the last acquisition trial, on day 6, the platform was removed, and a probe test was run. Animals were allowed to search for the platform for up to 60 s. The latency of the first entrance into the former platform area, the frequency of entrances, and the maximum velocity were analyzed. All experimental sessions were carried out between 9.00 am and 3.00 pm. A visual cue test was conducted 30 min after the probe test to assess motivation and visual ability.

*Assessment of visual responses -* The methods employed in [8] were used. The animals were box-anesthetized and maintained with isoflurane (respectively, 3% and 1%), placed on a stereotaxic frame, and the head fixed in place using ear bars. Body temperature was controlled using a heating pad and a rectal probe to maintain the animal’s body at 37°C. Local anesthesia was provided using subcutaneous lidocaine (2%) injection and eyes were protected with dexamethasone-based ointment (Tobradex, Alcon Novartis). The scalp was removed, and the skull carefully cleaned with saline. The skin was secured to the skull using cyanoacrylate. Then a thin layer of cyanoacrylate was poured over the exposed skull to attach a custom-made metal ring (9 mm internal diameter) centered over the binocular visual cortex. When the glue dried, a drop of transparent nail polish was spread over the area to ameliorate optical access. After surgery, the animals were placed in a heated box and monitored to ensure the absence of any sign of discomfort. Before any other experimental procedure, mice were left to recover for at least 36 h. During this period, paracetamol (5 mg/ml) was administered in the water as an analgesic therapy. Non-invasive transcranial intrinsic optical signal (IOS) recordings were performed under isoflurane anesthesia (0.5-1%) supplemented with an intraperitoneal injection of chlorprothixene hydrochloride (1.25 mg/kg). Images were obtained using an Olympus microscope (BX50WI). Red light illumination was provided by 8 red LEDs (625 nm, Knight Lites KSB1385-1P) attached to the objective (Zeiss Plan-NEOFLUAR 5x, NA: 0.16) using a custom-made metal LED holder. The animal was secured under the objective using a ring-shaped neodynium magnet mounted on an arduino-based 3D printed imaging chamber that also controls eye shutters and a thermostated heating pad [8]. Visual stimuli were generated using Matlab Psychtoolbox and presented on a gamma-corrected 9.7-inch monitor, placed 10 cm away from the eyes of the mouse. Contrast reversing Gabor patches (temporal frequency: 4 Hz, duration: 1 s) were presented in the binocular portion of the visual field (-10° to?+10° relative to the horizontal midline and -5° to?+50° relative to the vertical midline) with a spatial frequency of 0.03 cycles per degree, mean luminance 20 cd/m^2^, and a contrast of 90%. Visual stimulation was time locked with a 16 bit depth acquisition camera (Hamamatsu digital camera C11440) using a parallel port trigger. Interstimulus time was 14 s. Frames were acquired at 30 fps with a resolution of 512 × 512 pixels, low-pass filtered with a 2D average spatial filter (30 pixels, 117 μm^2^ square kernel), and down-sampled to 128 × 128 pixels. The signal was averaged for at least 120 trials and down-sampled to 10 fps. Fluctuations of reflectance (R) for each pixel were computed as the normalized difference from the average baseline (ΔR/R). For each recording, an image representing the mean evoked response was computed by averaging frames between 0.5 and 2.5 s after stimulation. A region of interest (ROI) was automatically calculated on the mean image of the response, by selecting pixels in the lowest 30% ΔR/R of the range between the maximal and minimal intensity value. Mean evoked responses were quantitatively measured as the average intensity inside the ROI. To weaken background fluctuations a manually selected polygonal region of reference (ROR) was subtracted. The ROR was placed where no stimulus response, blood vessel artifact, or irregularities of the skull were observed.

*Fear conditioning test* - The fear conditioning test was performed as described in [11]. The test occurred in a 30 x 24 x 21 cm sound-isolated operant chamber (Ugo Basile) in the presence of light (90 lux). Mice were trained and tested on 2 consecutive days. Training consisted of placing a mouse in the chamber and allowing it to explore freely for 2 min, after which a loud tone (85 dB, 2 kHz) was played for 20 s. A 0.75-mA foot shock was administered for the final two seconds of the loud tone. A second pairing of sound cue and shock was repeated after 1 min. The mouse was removed from the chamber 90 sec after the second shock. The animal was returned to the same chambers without a tone or shock, twenty-four hours after training, to test for context-dependent learning. At the end of 5 min in the contextual test, the mouse was returned to the home cage. One hour later, the mouse was placed in a novel chamber and allowed to explore freely for 2 min, after which a cue tone (85 dB, 2 kHz) was played for 1 min. Freezing behavior, defined as no movement except for respiration, was recorded using EthoVision 14XT software (Noldus Information Technology B.V.). The freezing behavior was measured when the animal returned to the testing chamber (contextual) and upon hearing the testing tone (cue) in the novel chamber.

*Non-invasive assessment of sleep and breathing pattern* - Hypnic and respiratory phenotypes of mice were assessed non-invasively with a validated technique based on whole-body plethysmography (WBP) as previously described [29, 30]. Mice were individually placed inside a modified 2-chamber WBP (PLY4223, Buxco, Wilmington), with the internal volume of the mouse chamber reduced to 0.97 L. Recordings of respiratory activity were performed for 8 hours during the light period. The wake-sleep states of mice were discriminated based on visual inspection of the WBP signal, using a validated procedure [29, 30]. This allowed the simultaneous evaluation of hypnic and respiratory phenotype, including the quantification of augmented breaths (sighs) and sleep apneas. Apneas and sighs were detected as breaths with duration (apneas) or tidal volume (sighs) greater than three times the average breath duration or tidal volume, respectively, for each mouse and sleep state. Because sighs often precede apneas, we further categorized apneas as post-sigh apneas if they followed a sigh by ≤8 s [31].

### Histological and Immunohistochemistry Procedures

For histological and immunohistochemistry analysis some animals from each experimental group were euthanized with iso?urane (2% in pure oxygen) and sacrificed through cervical dislocation. Brains were quickly removed and cut along the midline. Left hemispheres were processed for Golgi staining or ƴH2AX-immunolabeling, while right hemispheres were used for β-galactosidase staining or immunofluorescence as described below. All steps of sectioning, imaging, and data analysis were conducted blindly.

*Golgi staining* - Golgi staining was performed using the FD Rapid Golgi Stain TM Kit (FD NeuroTechnologies) according to the manufacturer’s instructions. Briefly, left hemispheres were immersed in the impregnation solution containing mercuric chloride, potassium dichromate, and potassium chromate, and stored at room temperature in darkness for 2-3 weeks. Hemispheres were cut with a microtome into 100-µm-thick coronal sections that were directly mounted onto gelatin-coated slides and were air dried at room temperature in the dark for an additional 2-3 days. After drying, sections were rinsed with distilled water and subsequently stained in the developing solution of the kit. A light microscope (Leica Mycrosystems) equipped with a motorized stage and focus control system and a color digital camera (Coolsnap-Pro; Media Cybernetics) were used for neuronal tracing and to take bright field images. Measurements were carried out using Image Pro Plus software (Media Cybernetics).

*Neuronal tracing* - Dendritic trees of Golgi-stained granule cells of the hippocampal dentate gyrus (DG) and pyramidal cells (basal dendrites) from layers II/III of the primary visual cortex (V1), were traced with a dedicated software that was custom-designed for dendritic reconstruction (Immagini Computer), interfaced with Image Pro Plus (Media Cybernetics). The dendritic tree (10-12 per animal) was traced live, at a final magnification of 500X, by focusing into the depth of the section. The operator starts tracing the branches that emerge from the cell soma and, after having drawn the first parent branch goes on with all the daughter branches of the next order in a centrifugal direction [32]. At the end of tracing, the program reconstructs the total dendritic length and the total number of branches.

*Spine density and morphology* - In Golgi-stained sections, spines of pyramidal neuron of CA1 and of layer II-III of V1 were acquired using a 100X oil immersion objective lens. Dendritic spine density was measured by manually counting the number of dendritic spines on apical dendrites of CA1 and V1 pyramidal neurons. In each mouse, 12-15 dendritic segments (segment length: 10 µm) from each zone were analyzed and spine density was expressed as total number of spines per 10 µm. Based on their morphology, dendritic spines can be divided into two different categories that reflect their state of maturation: immature spines (filopodia, thin and stubby-shaped) and mature spines (mushroom- and cup-shaped). The number of spines belonging to each class was counted and expressed as a percentage.

*ƴH2AX-immunolabeling* - Hemispheres were fixed by immersion in Glyo-Fixx (Thermo Electron Corp) for 24 h and embedded in paraffin. The forebrain was coronally sectioned into 8-μm-thick sections that were attached to poly-lysine-coated slides. One out of 12 sections from the hippocampal formation was stained using a rabbit polyclonal anti-phospho-ƴH2AX-Ser139 (1:1000; Abcam) antibody. Sections were retrieved with citrate buffer (pH 6.0) at 98°C for 40 min before incubation with the antibody and were processed as previously described [33]. Sections were incubated with a Cy3-conjugated anti-rabbit (1:200; Jackson Immunoresearch) secondary antibody and nuclei were counterstained with Hoechst 33342 (Sigma-Aldrich). Fluorescent images were taken using a Nikon Eclipse E600 microscope equipped with a Nikon Digital Camera DXM1200 (ATI system) under constant microscope settings. The intensity of Cy3 staining corresponding to the ƴH2AX signal was quantified in CA1 and CA3 layers and expressed as Optical Intensity/Area.

*β-galactosidase staining -* For β-galactosidase staining hemispheres were fixed by immersion in 4% PFA in 100 mM PBS, pH 7.4, stored in fixative for 48 h, kept in 20% sucrose for additional 24 h, and then frozen with dry ice. Hemispheres were cut with a freezing microtome into 30-μm-thick coronal sections that were serially collected. One out of 6 free-floating sections from the hippocampal formation and the primary somatosensory cortex (S1) were washed and incubated for 24 h with 1 mg/ml X-gal (Sigma-Aldrich) in the staining buffer supplemented with 5 mM potassium ferricyanide (Sigma-Aldrich) and 5 mM potassium ferrocyanide (Sigma-Aldrich) at 37°C. Stained samples were washed with PBS three times, dehydrated in ethanol of ascending purity (50, 70, 80, 90, and 100%, 2 min each) and mounted on gelatin-coated slides. Bright field images were taken under constant settings with a light microscope (Leica Mycrosystems) equipped with a motorized stage and focus control system and a color digital camera (Coolsnap-Pro; Media Cybernetics) and intensity of blue staining corresponding to the β-galactosidase signal was quantified in CA1, CA3, and from layers II/III to layer V of the S1, and expressed as Optical Intensity/Area.

*Immunofluorescence* - For immunofluorescence (i.e., cleaved caspase-3, NeuN, GFAP, S100β and TH) analyses hemispheres were fixed and cut in 30-μm-thick coronal sections as described above.

*NeuN staining* - For NeuN immunofluorescence, one out of every 6 sections from the hippocampal formation were incubated overnight with a mouse monoclonal anti-NeuN antibody (1:250; Millipore). The following day the sections were washed and incubated with a Cy3-conjugated anti-mouse IgG secondary antibody (1:200; Jackson Immunoresearch) and nuclei were counterstained with Hoechst 33342 (Sigma-Aldrich). Fluorescent images were acquired using a Nikon Eclipse TE600 microscope equipped with a Nikon Digital Camera DXM1200 ATI System (Nikon Instruments Inc.). The number of Hoechst positive nuclei in CA1 were manually counted using the point tool in Image Pro Plus (Media Cybernetics) and cell density was established as Hoechst positive cells/mm^3^. The density of neuron (NeuN) and pyknotic cells was established as cells/mm^3^.

*Cleaved caspase-3 staining* - For cleaved caspase-3 immunofluorescence, one out of 6 sections from the hippocampal formation and V1 were incubated overnight with a rabbit polyclonal anti-cleaved caspase-3 antibody (1:200; Cell Signaling Technology). The following day the sections were washed and incubated with an HRP-conjugated anti-rabbit secondary antibody (1:200; Jackson Immunoresearch). Detection was performed using the TSA Cyanine 3 Plus Evaluation Kit (Perkin Elmer) and nuclei were counterstained with Hoechst 33342 (Sigma-Aldrich). Fluorescent images were acquired using a Nikon Eclipse TE600 microscope equipped with a Nikon Digital Camera DXM1200 ATI System (Nikon Instruments Inc.). The density of apoptotic (cleaved caspase-3 positive) was established as cells/mm^3^.

*GFAP-staining -* Immunostaining for glial fibrillary acidic protein (GFAP) was performed on one out of 6 sections from the hippocampal formation using a mouse monoclonal anti-GFAP (1:400; Sigma-Aldrich) primary antibody, and a Cy3-conjugated anti-mouse IgG (1:200; Jackson Immunoresearch) secondary fluorescent antibody. Nuclei were counterstained with Hoechst 33342 (Sigma-Aldrich) and fluorescent images were acquired as above described. The number of GFAP-positive cells in the molecular layer of the hippocampal DG were manually counted and cell density was established as GFAP positive cells/mm^3^.

*S100*β-*staining* - Immunostaining for S100β protein was performed on one out of every 6 sections from the V1 cortex formation using a mouse monoclonal anti-S100β (1:200; Abcam) primary antibody, and a Cy3-conjugated anti-rabbit IgG (1:200; Jackson Immunoresearch) secondary fluorescent antibody. Nuclei were counterstained with Hoechst 33342 (Sigma-Aldrich) and fluorescent images were acquired as described above. The number of S100β-positive cells in the layers II/III of the V1 cortex were manually counted and cell density was established as S100β positive cells/mm^3^.

*TH-staining -* For tyrosine hydroxylase (TH) immunostaining one out of 4 coronal sections representing the medial and caudal striatum were stained using a rabbit polyclonal anti-TH (1:200; Boster Biological Technology) primary antibody, and an anti-rabbit IgG (1:200; Jackson Immunoresearch) secondary fluorescent antibody. Nuclei were counterstained with Hoechst 33342 (Sigma-Aldrich) and fluorescent images were acquired using a Nikon Eclipse TE600 microscope equipped with a Nikon Digital Camera DXM1200 ATI System (Nikon Instruments Inc.). The total number of TH positive neurons in the substantia nigra (SN) and ventral tegmental area (VTA) was estimated by multiplying the number counted in the series of sampled sections by the inverse of the section sampling fraction (section sampling fraction = 1/4).

*c-Fos, VGluT1 and Homer1bc immunofluorescence -* Immunofluorescence analyses were conducted as in [18]. Animals were anesthetized with an intraperitoneal injection of Zoletil/Xylazine (Sigma-Aldrich) and transcardially perfused with ice-cold PFA (4% in 100 mM PBS, pH 7.4). Brains were dissected out and kept in the same fixative solution overnight at 4 °C. The brains were then placed in cryoprotection solutions (10, 20, and 30% sucrose-PBS solutions), cut in coronal 30-μm-thick sections with a cryostat and stored at -20 °C in a solution containing 30% ethylene glycol and 25% glycerol until use. For immunofluorescence processing, cryosections were rinsed in PBS, placed in a solution containing 0.05% Triton X-100 and 10% normal donkey serum (NDS) in PBS for 1 h, followed by overnight incubation at room temperature with the following primary antibodies: rabbit polyclonal anti-cFos (1:500; Cell Signaling), rabbit polyclonal anti-Homer1bc (1:500; Synaptic System), rabbit polyclonal anti-VGluT1 (1:5000; Millipore). The following day, the sections were rinsed and incubated with suitable fluorescent secondary antibodies (1:1000; Jackson Immunoresearch). After several PBS washes, the sections were mounted on gelatin-coated glass slides and cover slipped with Dako fluorescence mounting medium (Dako Italia). For c-Fos cells density analyses, 8 serial optical sections were captured with a laser scanning confocal microscope (LSM5 Pascal; Zeiss) with a 40X objective (1.0 NA) using a 1-μm Z-step. Confocal images were acquired in the pyramidal layer of CA1 whereas micrographs spanning from the pial surface to the white matter were captured in S1 and V1 cortical areas as in [17]. Acquisition settings were kept constant throughout all imaging sessions, cells were manually counted on merged z-stacks using the point tool in ImageJ and their density was expressed as cells/mm^2^. Homer1bc puncta in the neuropil were analyzed on five serial optical sections (0.5 μm Z-step size) that were acquired from layers II/III and V of the S1 and V1 cortices with a confocal microscope (LSM5 Pascal; Zeiss) using a 100X objective (1.4 NA) and the pinhole set at 1 Airy unit. Synaptic puncta were manually counted using the point tool in ImageJ, included in the dataset if present in at least two consecutive optical sections. Homer1bc puncta are expressed as density (puncta/100 µm^2^). For the Homer1bc-VGluT1 co-apposition analysis, synaptic puncta in the neuropil from layers II/III and V of the S1 and V1 cortices were captured by using a laser scanning confocal microscope (LSM900; Zeiss) with a 63X objective (1.4 NA) and the pinhole set at 1 Airy unit on five serial optical sections (0.5 μm Z-step size). Co-appositions between postsynaptic Homer1bc and presynaptic VGluT1 signals were assessed by visual inspection in the three orthogonal planes and puncta were considered as co-apposed (i.e: putative synaptic contacts) when no black pixels were detected between signals. Synaptic puncta were manually counted and included in the dataset if present in at least two consecutive optical sections. The quantitation of Homer1bc-VGluT1 co-apposition is presented as percentage of VGluT1 puncta juxtaposed to Homer1bc.

**Figure 1.**
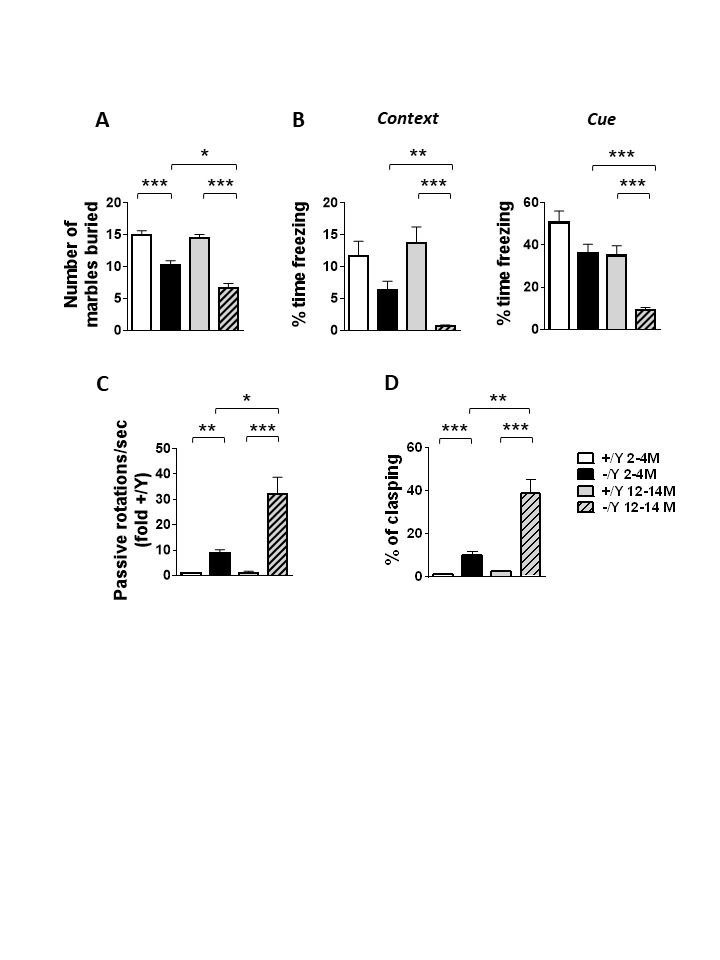
Age-dependent deterioration of behavior in *Cdkl5* KO mice. (A) Marble burying test in young adult *Cdkl5* -/Y (n = 38) and *Cdkl5* +/Y (n = 31) mice, and middle-aged *Cdkl5* -/Y (n = 29) and *Cdkl5* +/Y (n = 22) mice. (B) Fear conditioning paradigm, measuring time spent freezing in response to a mild footshock, upon the return to the testing chamber (context) and upon hearing the testing tone (cue) in adult *Cdkl5* -/Y (n = 29) and *Cdkl5* +/Y (n = 28) mice, and middle-aged *Cdkl5* -/Y (n = 22) and *Cdkl5* +/Y (n = 20) mice. (C) Frequency of passive rotations (rotations in which the mouse does not perform any coordinated movement but is passively transported on the rotating apparatus) in adult *Cdkl5* -/Y (n = 30) and *Cdkl5* +/Y (n = 19) mice, and middle-aged *Cdkl5* -/Y (n = 13) and *Cdkl5* +/Y (n = 10) mice. The mean frequency of passive rotations was expressed as fold of the wild-type counterparts of the same age. (D) Percentage of time spent hind-limb clasping during a 2-min interval in adult *Cdkl5* -/Y (n = 29) and *Cdkl5* +/Y (n =19) mice, and middle-aged *Cdkl5* -/Y (n = 27) and *Cdkl5* +/Y (n = 19) mice. Values represent mean ± SEM. *p< 0.05, **p< 0.01, ***p< 0.001 (Dunn’s test after Kruskal-Wallis).

### Western Blotting

Animals were euthanized with iso?urane (2% in pure oxygen) and sacrificed through cervical dislocation. The brain was quickly removed, and the hippocampal formation was homogenized in lysis buffer containing 20 mM Tris-HCl, pH 7.5, 1% SDS, 1 mM Na3VO4, 1 mM PMSF, 5% β-mercaptoethanol, and protease inhibitors (Sigma-Aldrich). Protein concentration was determined using the Bradford method [34]. Equivalent amounts (30 µg) of protein were subjected to electrophoresis on a 4-12% Mini- PROTEAN® TGX™ Gel (Bio-Rad) and transferred to a Hybond ECL nitrocellulose membrane (GE Healthcare Bio-Science). The following primary antibodies were used: rabbit polyclonal anti-GAPDH (1:5000, Sigma-Aldrich), rabbit polyclonal anti-XRCC5 (1:5000; Elabscience) and rabbit polyclonal anti-phospho-H2AX-Ser139 (1:1000; Abcam). An HRP-conjugated goat anti-rabbit IgG secondary antibody (1:5000; Jackson Immunoresearch) was used. Densitometric analysis of digitized images was performed using Chemidoc XRS Imaging Systems and Image LabTM Software (Bio-Rad).

### Statistical Analysis

Data from single animals represented the unity of analysis. Results are presented as mean ± standard error of the mean (± SE). Statistical analysis was performed using SPSS (version 23) or GraphPad Prism (version 6). All datasets were analyzed using the ROUT method (Q = 1%) to identify significant outliers and the Shapiro-Wilk test for normality. Datasets with normal distribution were analyzed for significance using a two-way analysis of variance (ANOVA) with genotype and age as factors. Post hoc multiple comparisons were carried out using the Fisher least significant difference (Fisher’s LSD) test. Datasets with nonparametric distribution were analyzed using the Kruskal-Wallis test. Post hoc multiple comparisons were carried out using Dunn’s multiple comparison test. For the learning phase of the MWM test, statistical analysis was performed using a repeated ANOVA. F-values for individual statistical analyses is reported in Supplementary Table 2. A probability level of p < 0.05 was considered to be statistically significant.

## RESULTS

### Behavioral phenotypes in middle-aged Cdkl5 -/Y mice

To determine the effect of age on physical parameters *in Cdkl5* KO mice, the body weight of *Cdkl5* +/Y and *Cdkl5* -/Y male mice was monitored longitudinally at 1, 2, 3, 6, and 12 months of age (Table 1). No significant weight differences were observed between genotypes in male *Cdkl5* KO mice, but a similar increment in the body weight/age trend was observed (Table 1).

Adult *Cdkl5* KO mice exhibit numerous behavioral deficits, including autistic-like behaviors, defects in motor coordination, and memory performance [9, 13, 35]. To establish whether these behavioral defects are retained or worsen at older development stages, we compared autistic-like, cognitive, and motor behaviors in adult (2- to 4-month-old) versus middle-aged (12- to 14-month-old) *Cdkl5* -/Y mice.

To evaluate the autistic-like phenotype, we analyzed exploration and interest in the environment using the marble burying test. Both adult and middle-aged *Cdkl5* -/Y mice buried a significantly lower number of marbles compared to their wild-type counterparts of the same age (Fig. 1A). Interestingly, while no age-dependent behavioral decline was observed in *Cdkl5* +/Y mice, middle-aged *Cdkl5* -/Y mice buried fewer marbles compared to younger *Cdkl5* -/Y mice (Fig. 1A), suggesting an age-dependent worsening of the autistic-like behavior.

Learning and memory were tested using a classic fear conditioning paradigm in which an innate freezing response was used as a readout for the ability of mice to learn and remember a context or an acoustic cue that is associated with an electric shock. We found a reduced (albeit not significant in younger animals) percentage of freezing time in *Cdkl5* -/Y mice during the re-presentation of both the context and the acoustic cue compared to their wild-type counterparts of the same age (Fig. 1B). Interestingly, middle-aged *Cdkl5* -/Y mice showed, in both paradigms, a reduced percentage of freezing time in comparison with younger *Cdkl5* -/Y mice (Fig. 1B), suggesting an age-dependent worsening of memory performance.

Hippocampus-dependent learning and memory were evaluated using the Morris Water Maze (MWM). Mice were tested for their ability to find a hidden platform for 5 days (learning phase) and were subjected to the probe test on day 6 (memory phase). Both young adult and middle-aged *Cdkl5* -/Y mice exhibit severe deficits in learning and memory (Supplementary Fig. 1A-C), but do not show differences in swimming speed (Supplementary Fig. 1D). After the hidden platform test, mice were tested with a visible platform MWM task. The aim of the visible platform test was to evaluate visual and motivational impairments in *Cdkl5* KO mice. While all *Cdkl5* +/Y mice of both ages quickly swam to the visible platform, 5.9% of young adult *Cdkl5* -/Y mice (1 out of a total of 17 mice) and, interestingly, 38% of middle-aged *Cdkl5* -/Y mice (8 out of a total of 21 mice) did not swim to the visible platform during the 60 sec of the trial. Since *Cdkl5* KO mice are characterized by impaired visual responses, we evaluated cortical visual responses using non-invasive transcranial intrinsic optical signal (IOS) imaging to compare visual impairment in young adult and middle-aged *Cdkl5* -/Y mice. Young adult and middle-aged *Cdkl5* -/Y mice showed a similar defect in visual responses compared with their wild-type counterparts of the same age (Supplementary Fig. 2), suggesting that the failure to reach the visual platform of 38% shown by middle-aged *Cdkl5* -/Y mice was not due to a worsening of visual responses, but might be attributed to less motivation regarding their environment.

We assessed the motor coordination of *Cdkl5* KO mice on an accelerating rotarod by evaluating the frequency of passive rotations (number of passive rotations/sec). Passive rotations, rotations in which the mouse does not perform any coordinated movements and is passively transported by the rotating apparatus, are a sign of motor coordination impairment [35]. Rotarod performance notably differed between *Cdkl5* +/Y and *Cdkl5* -/Y mice, with *Cdkl5* -/Y mice showing significantly more passive rotations (Fig. 1C). In particular, middle-aged *Cdkl5* -/Y mice showed a very high frequency of passive rotations, that was significantly greater than that of adult *Cdkl5* -/Y mice (Fig. 1C), indicating an age-dependent worsening of motor coordination.

Finally, in order to examine the effect of age on motor stereotypies, mice were tested for hind-limb clasping. While *Cdkl5* +/Y mice at both ages exhibited very little hind-limb clasping, adult *Cdkl5* -/Y mice spent about 1/4 of the test session (120 sec) in the clasping position (Fig. 1D). Again, middle-aged *Cdkl5* -/Y mice showed an age-dependent worsening of behavior with a large increase in clasping duration (Fig. 1D).

**Figure 2.**
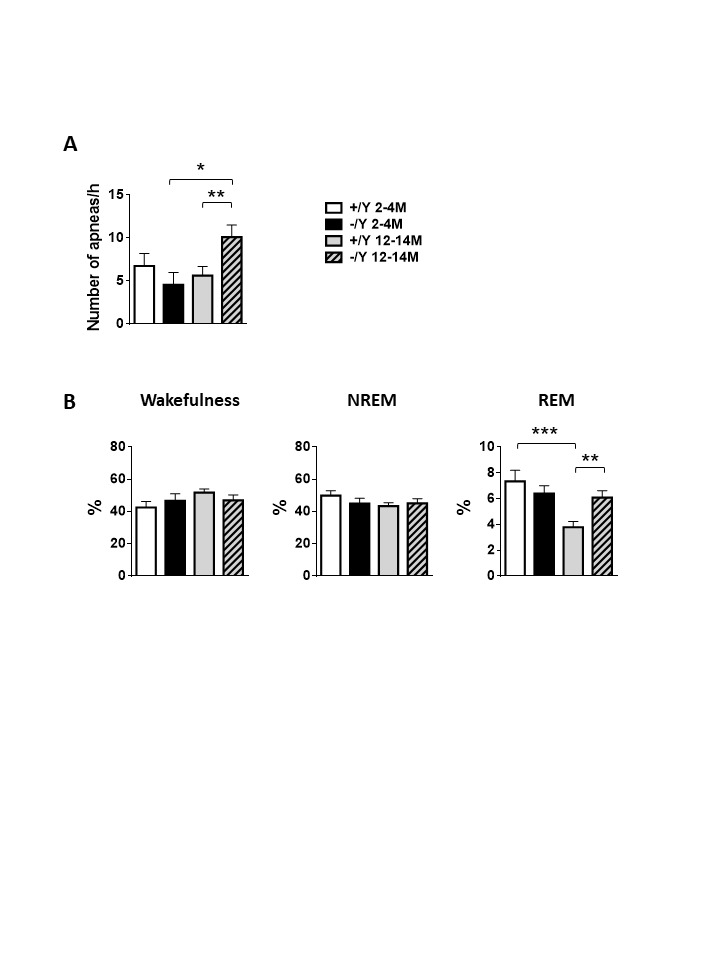
Sleep apnea and hypnic occurrence rate in *Cdkl5* KO mice. (A) Apnea occurrence rate during non-rapid-eye-movement sleep (NREM) sleep period in young adult *Cdkl5* -/Y (n = 8) and Cdkl5 +/Y (n = 9) mice, and middle-aged *Cdkl5* -/Y (n = 14) and *Cdkl5* +/Y (n = 14) mice. (B) Percentage of time spent in wakefulness, in NREM, and in rapid-eye-movement sleep (REM) during whole-body-plethysmography recordings in the same animals as in A. Values represent mean ± SEM. *p< 0.05, **p< 0.01, ***p< 0.001 (Fisher’s LSD test after two-way ANOVA).

**Figure 3.**
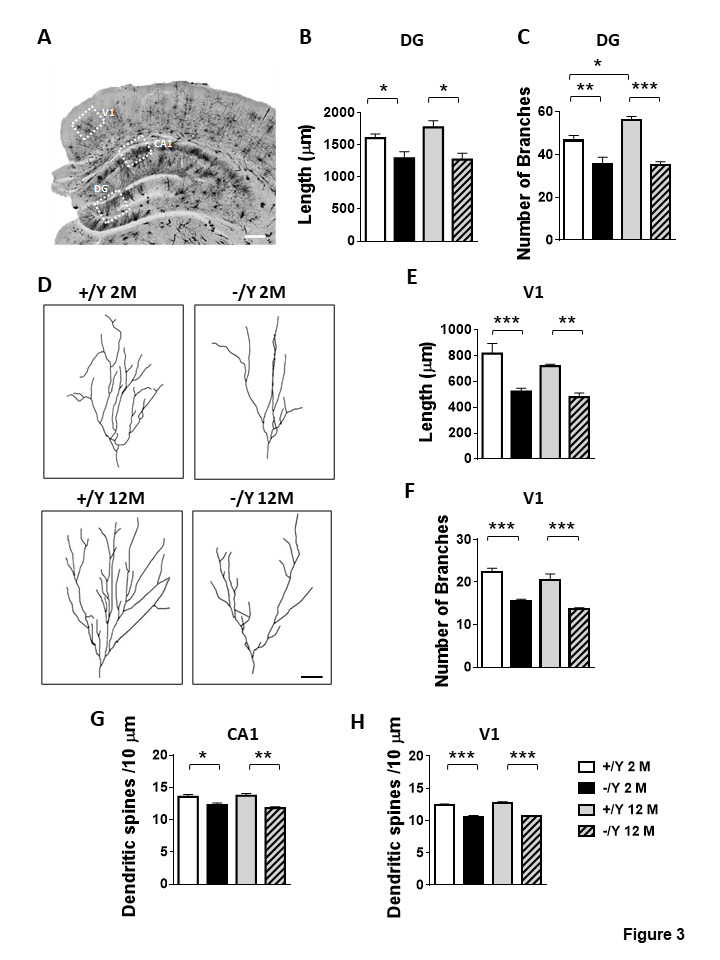
Impaired dendritic morphology in *Cdkl5* KO mice. (A) Example of a Golgi-stained brain slice. The dotted boxes highlight the brain regions of the hippocampal formation (granular layer (DG) and hippocampal CA1 region (CA1)) and the visual cortex (V1) where dendritic length and spine density and maturation were evaluated. Scale bar = 500 μm. (B-C) Mean total dendritic length (B) and mean number of dendritic segments (C) of Golgi-stained granule cells of the dentate gyrus (DG) in adult *Cdkl5* -/Y (n = 3) and *Cdkl5* +/Y (n = 3) mice, and middle-aged *Cdkl5* -/Y (n = 3) and *Cdkl5* +/Y (n = 3) mice. (D) Examples of the reconstructed dendritic tree of Golgi-stained mature granule neurons of one animal from each experimental group. Scale bar = 100 μm. (E-F) Mean total dendritic length (E) and mean number of basal dendritic segments (F) of Golgi-stained pyramidal neurons of the primary visual cortex (V1, layer II/III) in adult *Cdkl5* -/Y (n = 4) and *Cdkl5* +/Y (n = 4) mice, and middle-aged *Cdkl5* -/Y (n = 4) and *Cdkl5* +/Y (n = 4) mice. (G-H) Dendritic spine density (number of spines per 10 μm) on apical dendrites of pyramidal neurons of the CA1 layer of the hippocampus (G) and pyramidal neurons of V1 (layer II/III; H) from adult *Cdkl5* -/Y (n = 4 or 5, respectively) and *Cdkl5* +/Y (n = 4) mice, and middle-aged *Cdkl5* -/Y (n = 4) and *Cdkl5* +/Y (n = 4) mice. Values are represented as means ± SE. *p< 0.05, **p< 0.01, ***p< 0.001 (Fisher’s LSD after two-way ANOVA).

### Respiratory and hypnic phenotypes in middle-aged Cdkl5 -/Y mice

An abnormal breathing pattern, with a higher occurrence rate of sleep apneas, has been described in adult 4- to 6-month-old *Cdkl5* KO mice [30, 35], while at this age no changes in sleep pattern were observed between genotypes [30]. To determine whether *Cdkl5* -/Y breathing irregularities worsen with age and whether *Cdkl5* +/Y and *Cdkl5* -/Y mice show age-dependent sleep disturbances, we compared respiratory and hypnic phenotypes in young adult (2-month-old) versus middle-aged (12- to 14-month-old) mice, using non-invasive whole-body plethysmography (WBP). We found that the occurrence rate of sleep apneas of 2-month-old *Cdkl5* -/Y and *Cdkl5* +/Y mice did not differ, while middle-aged *Cdkl5* -/Y mice had more frequent apneas during non-rapid eye movement (NREM) sleep compared to *Cdkl5* +/Y mice (Fig. 2A). In further analyses, as a function of apnea type, middle-aged *Cdkl5* -/Y differed significantly from middle-aged *Cdkl5* +/Y in terms of higher occurrence rate of both post-sigh apneas (*Cdkl5* +/Y 4.312±3.588; *Cdkl5* -/Y 7.561±4.204 p = 0.0247) and the apneas that did not follow a sigh (*Cdkl5* +/Y 1.121±0.439; *Cdkl5* -/Y 2.544±1.894 p = 0.0122) during NREM sleep.

The sleep pattern of *Cdkl5* -/Y mice did not differ significantly from that of *Cdkl5* +/Y mice either in terms of percentage of wakefulness or of NREM sleep (Fig. 2B). In contrast, middle-aged *Cdkl5* -/Y mice spent a higher percentage of time in REM sleep compared to *Cdkl5* +/Y mice of the same age (Fig. 2B), suggesting an age-dependent sleep perturbation in the absence of CDKL5.

No significant differences in respiratory or hypnic variables were found between young (2-month-old) *Cdkl5* +/Y and *Cdkl5* -/Y mice (Fig. 2B).

### Dendritic abnormalities in middle-aged Cdkl5 -/Y mice

In order to establish whether the decline in behavioral performance in middle-aged *Cdkl5* -/Y mice is due to a deterioration of the dendritic pathology that characterizes *Cdkl5* KO mice, we analyzed dendritic architecture and spine density/maturation in the hippocampus and cortex of *Cdkl5* -/Y mice.

In Golgi-stained brain sections we examined the dendritic tree of granule cells of the dentate gyrus (DG) and pyramidal neurons of the primary visual cortex (V1) (Fig. 3A-F). Granule neurons of young adult and middle-aged *Cdkl5* -/Y mice displayed a similar lower dendritic length (Fig. 3B, D) in comparison with *Cdkl5* +/Y counterparts of the same age, due to a reduction in the number of branches (Fig. 3C, D). Similarly to granule neurons, pyramidal neurons of layer II/III of V1 exhibited dendritic hypotrophy, and a reduction in dendritic length (Fig. 3E) as well as in the number of branches (Fig. 3F).

**Figure 4.**
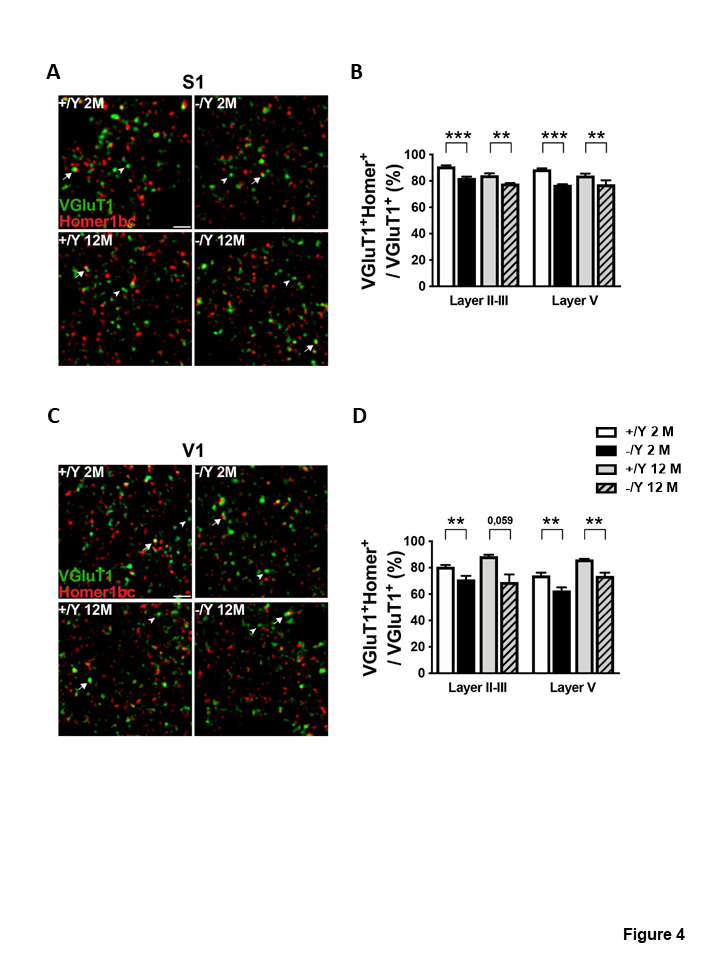
*Cdkl5* KO mice exhibit a decrease in the number of excitatory synaptic contacts. (A, C) Representative confocal micrographs of the neuropil in layer II-III of both S1 (A) and V1 (C) cortices showing immunofluorescence staining for VGluT1 (green) and Homer1bc (red) in young adult and middleaged *Cdkl5* +/Y and *Cdkl5* -/Y mice. Scale bar 5 μm. Arrows point to examples of VGluT1-Homer1bc coappositions. Arrowheads point to examples of solitary VGluT1+ terminals. (B, D) Quantitative analysis of the percentage of VGluT1+ terminals juxtaposed to Homer1bc+ in cerebral cortex from young adult *Cdkl5* -/Y (n = 4) and *Cdkl5* +/Y (n = 5) mice, and middle-aged *Cdkl5* -/Y (n = 3) and *Cdkl5* +/Y (n = 4) mice. Values are represented as means ± SE. **p<0.01, ***p<0.001 (Fisher’s LSD test after two-way ANOVA

Evaluation of dendritic spine density showed that both the pyramidal neurons of the hippocampus (CA1 field, Supplementary Fig. 3A) and cortex (V1) had a similar lower spine density in young adult and middle-aged *Cdkl5* -/Y mice in comparison with *Cdkl5* +/Y counterparts of the same age (Fig. 3G, H). Although, with age, regional differences in spine maturation are observed (Supplementary Fig. 3B), separate counts of different classes of dendritic spines revealed that the hippocampal and cortical pyramidal neurons of young adult and middle-aged *Cdkl5* -/Y mice had a higher percentage of immature spines and a reduced percentage of mature spines compared to age-matched *Cdkl5* +/Y mice (Supplementary Fig. 3B). A similar reduction in mature spine density in the cerebral cortex (V1 and S1) of *Cdkl5* -/Y mice at both ages was confirmed by the analysis of Homer1bc puncta density, a reliable postsynaptic marker of fully developed dendritic spines [36] (Supplementary Fig. 4). Finally, when we studied putative excitatory synaptic contacts, identified through the simultaneous labelling of both VGluT1 (excitatory presynaptic marker) and Homer1bc, we found that VGluT1^+^ terminals juxtaposed to Homer1bc^+^ puncta were significantly lower in the cerebral cortices of *Cdkl5* -/Y mice at both ages compared to their *Cdkl5* +/Y counterparts (Fig. 4).

### c-Fos expression in middle-aged Cdkl5 -/Y mice

**Figure 5.**
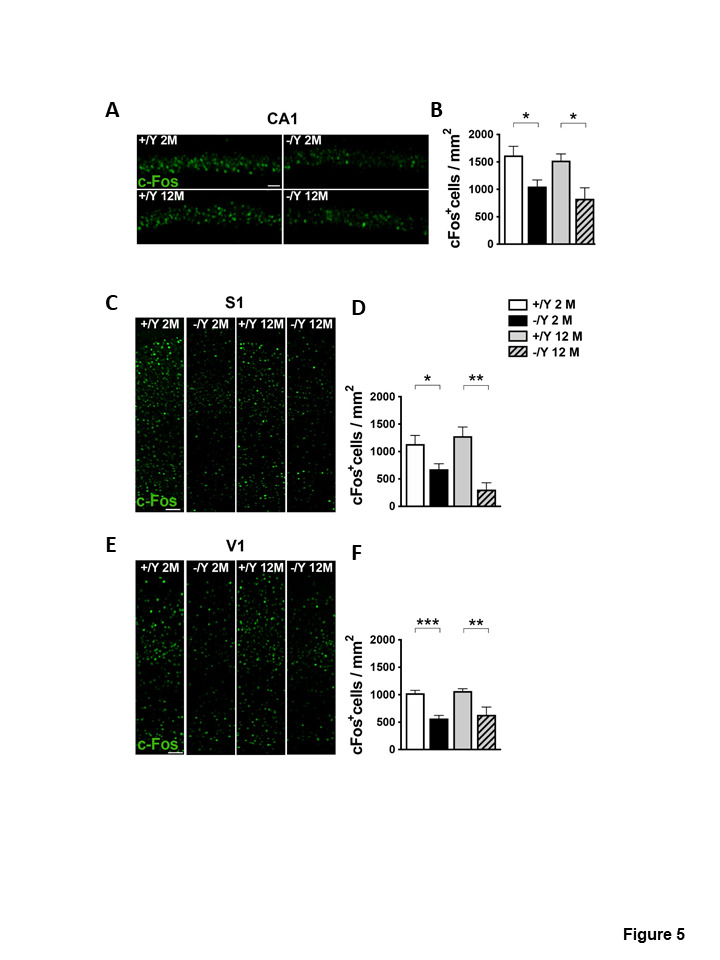
*Cdkl5* KO mice exhibit a reduction of the number of c-fos. (A, C, E) Representative examples of c-Fos staining obtained from the pyramidal cell layer of the hippocampal CA1 (A), and from the primary somatosensory S1 (C) and primary visual cortices V1 (E) of young adult and middle-aged *Cdkl5* +/Y and *Cdkl5* -/Y mice. Scale bar = 50μm. (B) Quantification of the density of c-Fos immunoreactive cells in the CA1 layer from young adult *Cdkl5* +/Y (n = 6) and *Cdkl5* -/Y (n = 7) mice, and middle-aged *Cdkl5* +/Y (n = 3) and *Cdkl5* -/Y (n = 4) mice. (D) Quantitative analysis of the density of c-Fos positive cells in the S1 cortex of young adult *Cdkl5* +/Y (n = 8) and *Cdkl5* -/Y (n = 10) mice, and middle-aged *Cdkl5* +/Y (n = 4) and *Cdkl5* - /Y (n = 3) mice. (F) Quantification of c-Fos positive cells in the V1 cortex of young adult *Cdkl5* +/Y (n = 5) and *Cdkl5* -/Y (n = 5) mice, and middle-aged *Cdkl5* +/Y (n = 3) and *Cdkl5* -/Y (n = 3) mice. Values are represented as means ± SE. *p<0.05, **p<0.01, ***p<0.001 (Fisher’s LSD test after two-way ANOVA).

We have previously shown that in 6- to 8-week-old *Cdkl5* -/Y mice the reduction in c-Fos expression detected by immunohistochemistry is a robust cortical endophenotype, most likely indicating less neuronal activation [17, 18]. To assess whether defects in the expression levels of the immediate early gene c-Fos parallel the decline in behavioral performance in middle-aged *Cdkl5* -/Y mice, we evaluated the number of c-Fos positive neurons in the CA1 field of the hippocampus as well as in the S1 and V1 cortices of both 2- and 12-month-old *Cdkl5* KO mice. As shown in Figure 5 the number of c-Fos immunoreactive cells is clearly reduced in these three brain areas (Fig. 5 A, C and E) of *Cdkl5* -/Y mice at both ages. In agreement with this, the quantitative analyses revealed a statistically significant reduction in the density of c-Fos positive neurons between genotypes but no age differences (Fig. 5B, D and F), indicating that a reduction in c-Fos expression is not dependent on the age of *Cdkl5* -/Y mice.

### Neuronal survival in middle-aged Cdkl5 Y/- mice

**Figure 6.**
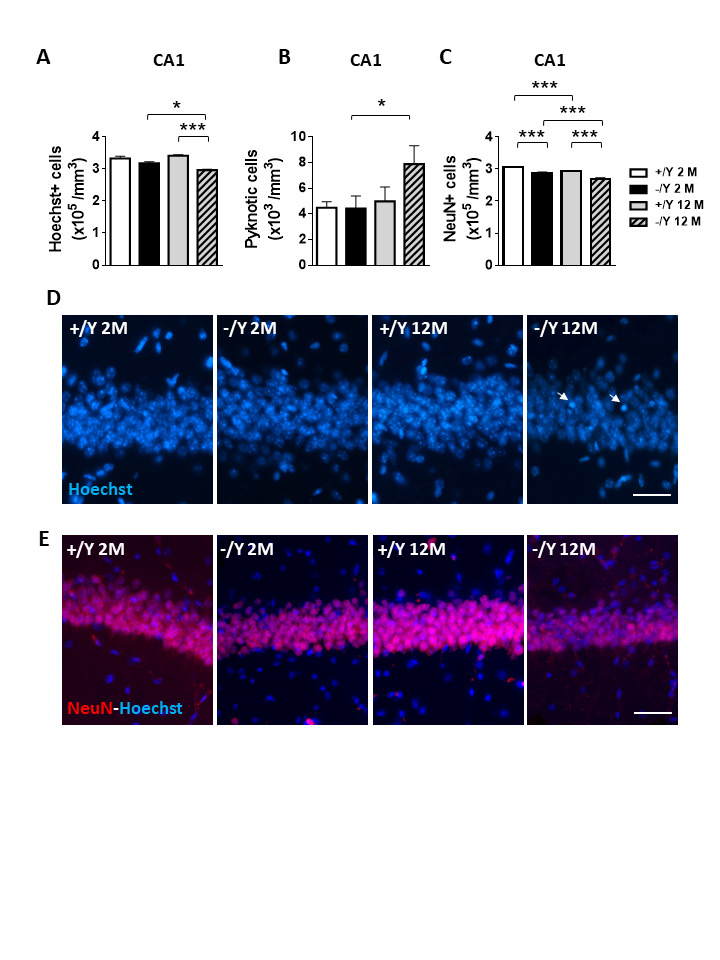
Reduced neuronal survival in *Cdkl5* KO mice. (A) Quantification of Hoechst-positive cells in CA1 of hippocampal sections from young adult *Cdkl5* +/Y (n = 4) and *Cdkl5* -/Y (n = 4) mice, and middleaged *Cdkl5* +/Y (n = 4) and *Cdkl5* -/Y (n = 4) mice. (B) Quantification of pyknotic cells in the CA1 layer of the hippocampus from mice as in B. (C) Quantification of NeuN-positive cells in CA1 of hippocampal sections from young adult *Cdkl5* +/Y (n = 4) and *Cdkl5* -/Y (n = 4) mice, and middle-aged *Cdkl5* +/Y (n = 5) and *Cdkl5* -/Y (n = 4) mice. (D) Representative fluorescence images of cell layers in the hippocampal CA1 region from young adult and middle-aged *Cdkl5* +/Y and *Cdkl5* -/Y mice. Arrows indicate pyknotic nuclei. Scale bar = 100 μm. E) Representative fluorescence images of sections immunopositive for NeuN (red) and counterstained with Hoechst (blue) in the hippocampal CA1 region of one animal from each group. Scale bar = 100 μm. Values are represented as means ± SE. *p<0.05, **p<0.01, ***p<0.001 (Fisher’s LSD test after two-way ANOVA).

In order to evaluate whether a loss of *Cdkl5* affects neuronal survival rate in elderly mice, we first evaluated cell density in the CA1 layer of the hippocampus (Fig. 6 A-C). We found that middle-aged *Cdkl5* -/Y mice showed a reduced cell number and an increase in the number of pyknotic nuclei in the CA1 layer in comparison with age-matched *Cdkl5* +/Y mice (Fig. 6A, B). To confirm the reduced neuronal survival rate, we counted the number of NeuN-positive pyramidal neurons in the CA1 layer. We found a reduction in NeuN-positive neurons in both young adult and middle-aged *Cdkl5* -/Y mice compared to age-matched *Cdkl5* +/Y mice (Fig. 6C). Importantly, although a reduction in NeuN-positive neurons was present in middle-aged *Cdkl5* +/Y mice, the reduction was significantly higher in middle-aged *Cdkl5* -/Y mice (Fig. 6C).

**Figure 7.**
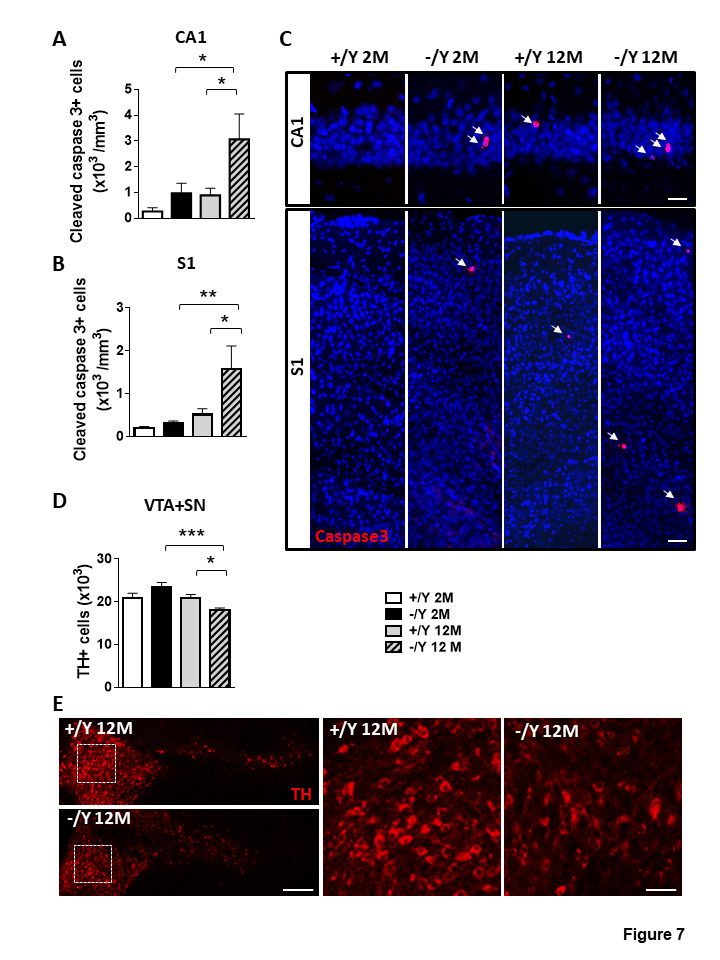
Increased neuronal cell death in *Cdkl5* KO mice. (A-B) Quantification of cells positive for cleaved caspase-3 in the CA1 layer of the hippocampus (A) and in layer II/III of the somatosensory cortex (S1, B) from young adult *Cdkl5* +/Y (n = 4) and *Cdkl5* -/Y (n = 4) mice, and middle-aged *Cdkl5* +/Y (n = 4) and *Cdkl5* -/Y (n = 4) mice. (C) Representative images, one from each group, of cells, in the hippocampal CA1 region (upper panel) and in layer II/III of the somatosensory cortex (S1 , lower panel), immunopositive for cleaved caspase-3 (red) and stained with Hoechst (blue). Scale bar = 50 μm. (D) Quantification of the total number of TH-positive neurons in the substantia nigra (SN) and ventral tegmental area (VTA) from young adult *Cdkl5* +/Y (n = 5) and *Cdkl5* -/Y (n = 4) mice, and middle-aged *Cdkl5* +/Y (n = 4) and *Cdkl5* - /Y (n = 5) mice. (E) Representative images of tyrosine hydroxylase (TH) immunofluorescence staining in the VTA and SN of middle-aged *Cdkl5* +/Y and *Cdkl5* -/Y mice. Scale bar = 100 μm. The dotted boxes indicate the VTA region shown at a higher magnification in the right panel. Scale bar = 40 μm. Values are represented as means ± SE. *p<0.05, **p<0.01, ***p<0.001 (Fisher’s LSD test after two-way ANOVA).

Apoptotic cell death was evaluated by counting the number of cleaved caspase-3-positive cells (Fig. 7A-C). An increase in cell death was already present in young adult *Cdkl5* -/Y mice, but it was significantly higher in middle-aged *Cdkl5* -/Y mice (Fig. 7A, C). Similarly, we found increased apoptosis in the S1 cortex of middle-aged *Cdkl5* -/Y mice (Fig. 7B, C).

Since reactive gliosis is a hallmark of neurodegeneration [37], we evaluated the number of GFAP-positive cells in the molecular layer of the hippocampal DG of adult and middle-aged *Cdkl5* KO mice. We observed a higher number of GFAP-positive cells in middle-aged *Cdkl5* -/Y mice in comparison with *Cdkl5* +/Y mice (Supplementary Fig. 5A, B), an increase that was not present in young adult *Cdkl5* -/Y mice (Supplementary Fig. 5A, B). In contrast, an increase in the number of S100β-positive astrocytes was present in the cortex of both young adult and middle-aged *Cdkl5* -/Y mice compared to age-matched *Cdkl5* +/Y mice (Supplementary Fig. 5C, D). The increase was significantly higher in middle-aged *Cdkl5* -/Y mice compared to young adult *Cdkl5* -/Y mice (Supplementary Fig. 5C, D).

**Figure 8.**
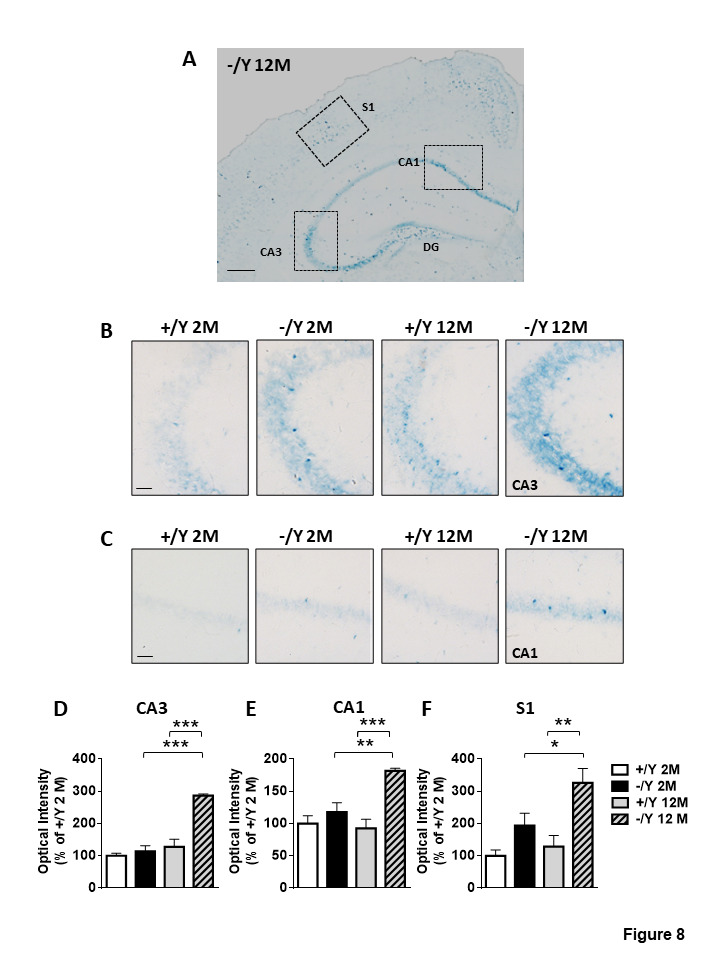
Senescence-associated-β-galactosidase (SA-β-gal) activity in *Cdkl5* KO mice. (A) Representative image at low magnification of a hippocampus of a middle-aged *Cdkl5* -/Y mouse. Scale bar = 500 μm. The dotted boxes indicate the regions used for quantification in (D-F). (B-C) Higher magnification shows representative images of SA-β-gal staining in the hippocampal CA3 (B) and CA1 (C) region from adult and middle-aged *Cdkl5* +/Y and *Cdkl5* -/Y mice. Scale bar = 50 μm. (D-F) Quantification of SA-β-gal intensity in CA3 (D) and CA1 (E) hippocampal layers, and in layer II/III of the S1 cortex (F) from young adult *Cdkl5* +/Y (n = 3) and *Cdkl5* -/Y (n = 4) mice, and middle-aged *Cdkl5* +/Y (n = 4) and *Cdkl5* -/Y (n = 3) mice. Values are represented as means ± SE. *p< 0.05, **p< 0.01, ***p< 0.001 (Fisher’s LSD test after two-way ANOVA).

**Figure 9.**
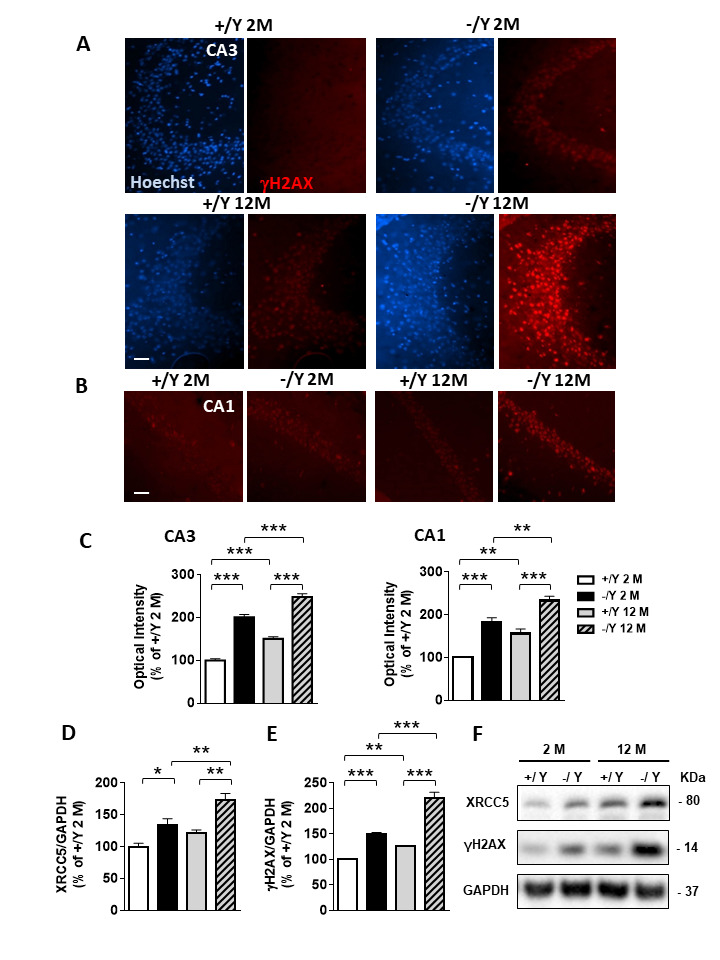
Increased DNA damage in the hippocampus of *Cdkl5* KO mice. (A-B) Representative fluorescent images of the CA3 (A) and CA1 (B) hippocampal regions of adult and middle-aged *Cdkl5* +/Y and *Cdkl5* -/Y mice, immunostained for γH2AX and counterstained with Hoechst. Scale bar = 500 μm (C) Quantification of γH2AX nuclear signal intensity in the CA3 and CA1 pyramidal layer of young adult *Cdkl5* +/Y (n = 3) and *Cdkl5* -/Y (n = 3) mice, and middle-aged *Cdkl5* +/Y (n = 4) and *Cdkl5* -/Y (n = 3) mice. (D) Western blot analysis of γH2AX levels normalized to GAPDH levels in the hippocampus of young adult *Cdkl5* +/Y (n = 4) and *Cdkl5* -/Y (n = 5) mice, and middle-aged *Cdkl5* +/Y (n = 3) and *Cdkl5* -/Y (n = 3) mice. (E) Western blot analysis of XRCC5 levels normalized to GAPDH levels in the hippocampus of young adult *Cdkl5* +/Y (n = 4) and *Cdkl5* -/Y (n = 5) mice, and middle-aged *Cdkl5* +/Y (n = 4) and *Cdkl5* -/Y (n = 3) mice. (F) Immunoblot images of γH2AX, XRCC5, and GAPDH levels in protein extracts from one animal of each experimental group. Data in Western blot analysis are expressed as a percentage of the values compared to young adult *Cdkl5* +/Y mice. Values are represented as means ± SE. *p< 0.05 and **p< 0.01, ***p<0.001 (Fisher’s LSD test after two-way ANOVA).

To determine whether impaired neuronal survival could be extended to other brain regions and other neuronal subtypes, we evaluated the total number of dopaminergic neurons in the substantia nigra (SN) and ventral tegmental area (VTA) of the midbrain (Fig. 7D, E). We found that the number of tyrosine hydroxylase (TH)-positive cells was significantly lower in middle-aged *Cdkl5* -/Y mice compared to *Cdkl5* +/Y mice (Fig. 7D, E), while there was no difference in the number of TH-positive cells between young adult *Cdkl5* -/Y and *Cdkl5* +/Y mice (Fig. 7D, E).

### Neuronal senescence in middle-aged Cdkl5 -/Y mice

Cells with features of senescence have been detected in the context of brain aging and neurodegenerative disease [38]. To evaluate senescent cells in *Cdkl5* KO mice we used a common marker: intracellular level of β-galactosidase (SA-β-GAL) activity. Interestingly, we observed an intense staining of the whole hippocampus (Fig. 8A), and in particular the CA3 layer, in middle-aged *Cdkl5* -/Y mice (Fig. 8B, D), but a low level of staining in their wild-type (*Cdkl5* +/Y) counterparts and in young adult *Cdkl5* -/Y and +/Y mice (Fig. 8B, D). Similarly, middle-aged *Cdkl5* -/Y mice showed a higher level of SA-β-GAL staining in the CA1 layer of the hippocampus and in S1, compared to *Cdkl5* +/Y mice of the same age (Fig. 8C, E, F).

### DNA damage in middle-aged Cdkl5 -/Y mice

Defective DNA repair with accumulation of unrepaired DNA is an underlying cause of brain aging [39]. Interestingly, a recent finding suggests that CDKL5 plays a role in the regulation of the DNA repair process [23]. To determine whether reduced neuronal survival and increased senescence in *Cdkl5* -/Y mice is paralleled by an increased DNA damage response, we analyzed the presence of DNA double-strand breaks in hippocampal neurons of *Cdkl5* KO mice by assaying for the levels of phospho-histone H2AX (γH2AX). We found that both young adult and middle-aged *Cdkl5* -/Y mice showed a higher level of γH2AX immunostaining in comparison with *Cdkl5* +/Y mice of the same age (Fig. 9A-C). Moreover, although γH2AX levels increased with age in both *Cdkl5* +/Y and *Cdkl5* -/Y mice (Fig. 9A-C), middle-aged *Cdkl5* -/Y showed the highest levels of γH2AX immunostaining (Fig. 9A-C) in both layers that were analyzed: CA3 and CA1. Similarly, the expression levels of γH2AX and of the double-strand break (DSB)-repair protein, XRCC5, analyzed using Western blot analyses, were higher in hippocampal extracts of middle-aged *Cdkl5* -/Y mice compared to young *Cdkl5* -/Y mice and to *Cdkl5* +/Y mice of the same age (Fig. 9D-F), suggesting increased DNA damage in the absence of *Cdkl5* as a function of age.

## DISCUSSION

Although the clinical aspects associated with *CDKL5* mutations are well described in children, adults with CDD are still under-characterized. An age-related analysis of the phenotype in CDD is an important research priority for a better understanding of the time-related progression of the multiple symptoms that may be involved in this pathology, thus allowing them to be targeted more effectively using appropriate treatment strategies. Although very little is known regarding the life expectancy of CDD patients, there are currently no reported cases of CDD patients older than middle-aged (40-45 years; [7]), suggesting a shortened lifespan. Therefore, based on the reported CDD human cases (most of them are under 18 years of age; [7]), we decided to analyze the phenotype of young/adult *Cdkl5* KO mice (2-4 months in the mouse corresponding to 6-15 years in humans; [40]) in comparison with middle-aged *Cdkl5* KO mice (12-14 months in the mouse corresponding to 40-45 years in humans; [40]). In this study, we report an age-dependent decline in motor, cognitive, and social behaviors in *Cdkl5* KO mice. We found a decreased neuronal survival in middle-aged *Cdkl5* KO mice that was paralleled by an increase in neuronal senescence and DNA repair protein levels. Our findings suggest that the absence of CDKL5 accelerates neuronal senescence by triggering irreparable DNA damage, and thereby provide evidence that CDKL5 may play a fundamental role in neuronal survival during brain aging.

### Worsening of the behavioral phenotype with age in Cdkl5 KO mice

Autistic-like (ASD-like) features, including disinterest in the surrounding environment, intellectual disability, and motor dysfunction are prominent features of CDKL5 deficiency and have been commonly described in *Cdkl5* KO mouse models. Our finding that middle-aged *Cdkl5* -/Y mice showed a further decreased interest in the environment (reduced marble burying and failure to reach the visible platform in the Morris water maze paradigm) in comparison to young adult *Cdkl5* -/Y mice, indicates an age-dependent worsening of ASD-like features in the absence of CDKL5.

Importantly, cognitive abilities, such as memory acquisition, were characterized by a further deterioration in middle-aged *Cdkl5* -/Y mice. Likewise, middle-aged *Cdkl5* -/Y mice showed an increased frequency of uncoordinated rotations on the rotating rod, and a longer clasping time, indicating a worsening in motor coordination and stereotypies. Since we did not find a similar behavioral deterioration in middle-aged wild-type mice, the behavioral worsening in older *Cdkl5* -/Y mice indicates a CDKL5-dependent deterioration of the neurological phenotype.

Our findings that complex behaviors such as sleep, and breathing are age-dependent in the *Cdkl5* KO mouse support our hypothesis of a worsening of the behavioral phenotype with age in CDD. We previously found that there is a difference in breathing during sleep between *Cdkl5* KO and wild-type adult mice (4 to 6 months-old). In particular, we demonstrated that *Cdkl5* -/Y adult mice showed more frequent sleep apneas compared to *Cdkl5* +/Y [30] mice, and that, at least in adult (4- to 6- month-old) mice, sleep apneas are a core feature of CDKL5 disorder and a respiratory biomarker of CDKL5 deficiency. In the present study, we showed that young (2-month-old) mice have a completely normal sleep and breathing phenotype; on the contrary, we found different changes in sleep and breathing phenotype in *Cdkl5* -/Y at 12 months of age, with a significant increase in NREM sleep apneas compared to *Cdkl5* -/Y. These data confirm the results that we previously found in 4-6 month-old *Cdkl5* -/Y mice [30]. Furthermore, the present study demonstrated that at the age of 12 months *Cdkl5* -/Y mice also show a significant increase in post-sigh apneas, which had not significantly increased at the age of 4 to 6 months. This suggests the presence of a progressive age-dependent derangement of the respiratory phenotype in *Cdkl5* -/Y mice.

Moreover, REM sleep time in middle-aged *Cdkl5* -/Y mice is increased compared to that of control mice. This is interesting, because REM sleep disturbances and increased REM sleep duration have been considered as biological markers of depression [41], and REM sleep represents an element of the response to chronic stress [42]. Thus, this specific modification of the hypnic phenotype that appears in 12-month-old *Cdkl5* -/Y mice may represent an index of age-dependent behavioral dysfunction.

### Synaptic and dendritic pathology does not worsen with age in Cdkl5 KO mice

Changes in neuronal morphology have consistently been reported in several *Cdkl5* KO mouse models [9, 10]. Neuronal tracing of Golgi-stained, or DCX-stained cells in juvenile and adult *Cdkl5* KO mice showed a reduction in dendritic length in cortical and hippocampal pyramidal neurons, as well as in hippocampal granule cells [9, 13-15]. Furthermore, *Cdkl5* KO mice show changes in the maturation and stability, as well as in the density, of dendritic spines in several brain structures (somatosensory, visual and perirhinal cortex, hippocampus, and thalamus) [13, 15-17, 43, 44]. Dendritic pathology is a possible substrate for mental retardation in different conditions. In CDD mouse models, the pathogenesis of dendritic abnormalities is distinctive and appears to correlate with the cognitive profile [9, 13-15]. Our finding that defects in neuronal maturation as well as in synapse development and configuration do not worsen with age, indicates that the behavioral decline in middle-aged *Cdkl5* -/Y mice is not caused by a worsening of the dendritic pathology.

### Increased neuronal cell death in middle-aged Cdkl5 KO mice

The role of CDKL5 in neuronal survival has recently been described [22]. In particular, *Cdkl5* KO neurons have been demonstrated to be more prone to cell death since they are more vulnerable to neurotoxic/excitotoxic stimuli [22]. During aging, neuronal cells are subjected to increased oxidative stress, perturbed energy homeostasis and mutations in nucleic acids. We assume that these alterations make *CDKL5* KO neurons more vulnerable during aging, as suggested by the increased cell death found in the brains of middle-aged *Cdkl5* -/Y mice. Death of neurons adversely affects both the pre- and post-synaptic neurons with which they communicate. Therefore, a small neuronal degeneration, such as that observed in older *Cdkl5* KO mice, may cause a serious brain dysfunction for a domino-like effect. As a neurochemical marker that is putatively associated with the increased neurodegeneration, we found an astrocytic hyperactivity in the brains of older *Cdkl5* KO mice. Since the astrocytic marker S100β is expressed by a subtype of mature astrocytes that ensheath blood vessels, and by NG2-expressing cells, which are precursors to oligodendrocytes [45-47], we cannot exclude the possibility that other alterations, regarding blood-brain barrier permeability or the axonal myelination, may occur in elderly *Cdkl5* KO mice.

Unlike neurodegenerative disorders, most of which are characterized by the degeneration of a specific neuronal population, the absence of CDKL5 did not reveal a selective neuronal vulnerability. Our findings show that neuronal death occurs in different brain regions (hippocampus, cortex, and basal ganglia) and affects different neuronal populations in older *Cdkl5* KO mice, supporting the general decline in behavior, including the impairment of motor and cognitive functions. While the decline in memory performance in middle-aged *Cdkl5* KO mice correlates well with decreased hippocampal neuron survival, we can speculate that motor impairment may depend on the loss of TH-positive neurons.

### Increased neuronal senescence and damaged DNA response with age in Cdkl5 KO mice

As an indicator of senescence (SA-β-GAL) is more active in a variety of cell types of the *Cdkl5* KO mouse brain with age, it is tempting to speculate that senescent cells, which are more susceptible to death, contribute to negatively influence behavior in older *Cdkl5* KO mice. Senescence is associated with increased amounts of damaged DNA in neurons which may result from impaired DNA repair systems [39]. Genetic diseases caused by defects in DNA damage repair (DDR) are associated with premature aging syndromes with neurodegenerative phenotypes; this is consistent with the impairment of these systems playing a role in normal aging of the nervous system. Accordingly, we found that loss of Cdkl5 promotes both premature cellular senescence and endogenous DDR signaling by increasing γH2AX and XRCC5 levels in the brain of older *Cdkl5* KO mice, suggesting that accumulation of nuclear DNA damage stimulates the activity of proteins that hold key functional roles in DNA repair and, subsequently, triggers the initiation of cellular senescence.

Since several lines of evidence indicate that the activity of kinases, including MAP kinases, decreases during aging [48-50], it can be speculated that an age-dependent decline of CDKL5 activity might be implicated in the physiological neuronal senescence. Although, to date, very little is known regarding the post-translational modifications of CDKL5 or its molecular targets, the presence of a TXT activation motif of MAP kinases in its sequence and of a putative signal peptidase I serine active site, suggests that CDKL5 activity might be affected by post-translational modifications, according to neuronal maturation or age.

In conclusion, our study indicates that the upregulated neuronal vulnerability due to reduced DNA repair and increased cellular senescence might play a role in neuronal dysfunction and impaired behavior in older *Cdkl5* KO mice. Based on our results, a worsening of the clinical symptoms in adult and elderly CDD patients seems predictable.

## Supplementary Materials

The Supplemenantry data can be found online at: www.aginganddisease.org/EN/10.14336/AD.2020.0827.


