# Supplementary material for: Age-Related Cognitive and Motor Decline in a Mouse Model of CDKL5 Deficiency Disorder is Associated with Increased Neuronal Senescence and Death
Source: Aging Dis. 2021 Jun 1;12(3):764–85. doi: 10.14336/AD.2020.0827 (PMC8139207; doi:10.14336/AD.2020.0827)
Supplement: Supplementary file 1 [file AD-12-3-764-s.pdf]

SUPPLEMENTARY DATA

Age-Related Cognitive and Motor Decline in a Mouse Model of CDKL5 Deficiency Disorder is Associated with Increased Neuronal Senescence and Death

Laura Gennaccaro[1], Claudia Fuchs[1,#], Manuela Loi[1,#], Riccardo Pizzo[2], Sara Alvente[1], Chiara Berteotti[1], Leonardo Lupori[3,4], Giulia Sagona[4,5,6], Giuseppe Galvani[1], Antonia Gurgone[2], Alessandra Raspanti[2], Giorgio Medici[1], Marianna Tassinari[1], Stefania Trazzi[1], Elisa Ren[1], Roberto Rimondini[7], Tommaso Pizzorusso[3,4,5], Giovanna Zoccoli[1], Maurizio Giustetto[2,8], Elisabetta Ciani[1,*]

© 2020. Gennaccaro L et al. Published online at http://www.aginganddisease.org/EN/10.14336/AD.2020.0827

SUPPLEMENTARY DATA

Supplementary Table 1. Number of mice included in the analyses**.**

	Cdkl5 +/Y		*Cdkl5* -/Y	
**AGE (month)**	**2-4**	**12-14**	**2-4**	**12-14**
Marble Burying	31	22	38	29
Fear conditioning test	28	20	29	22
Morris water maze (MWM)	18	11	17	21
Non-invasive transcranial intrinsic optical signal (IOS)	9	8	6	7
Accelerating Rotarod Assay	19	10	30	13
Hind-limb Clasping	19	19	29	27
Non-invasive whole-body plethysmography (WBP)	9	14	8	14
Length/Number of Branches DG	3	3	3	3
Length/Number of Branches V1	4	4	4	4
Spine density/Maturation CA1	4	4	4	4
Spine density/Maturation V1	5	4	4	4
VGluT1-Homer1bc puncta S1	5	4	4	3
VGluT1-Homer1bc puncta V1	5	4	4	3
c- Fos+ cells CA1	6	7	3	4
c- Fos+ cells S1	8	10	4	3
c- Fos+ cells V1	5	5	3	3
Density CA1/Pyknotic cells	4	4	4	4
NeuN+ cells	4	4	5	4
Cleaved caspase-3+ cells	4	4	4	4
GFAP+ cells	4	4	4	4
S100+ cells	4	4	4	4
Number of TH+ cells	5	4	4	5
SA-β-GAL activity	3	4	4	3
γH2AX intensity	3	4	3	3
Western Blot (XRCC5/GAPDH)	4	4	5	3
Western Blot (γH2AX/GAPDH)	4	3	5	3
Homer1bc puncta S1	5	5	4	3
Homer1bc puncta V1	3	4	4	3

© 2020. Gennaccaro L et al. Published online at http://www.aginganddisease.org/EN/10.14336/AD.2020.0827

SUPPLEMENTARY DATA

Supplementary Table 2. Two-way ANOVA tabular results.

	**Genotype**	**Age**	**Genotype*Age**
Total Apneas	F(1,41)=0.6699 p=0.4178	F(1,41)=2.542 p=0.1185	F(1,41)=5.780 p=0.0208
Wakefulness	F(1,41)=0.0077 p=0.9304	F(1,41)=1.930 p=0.1722	F(1,41)=1.678 p=0.2024
NREM	F(1,41)=0.2936 p=0.5909	F(1,41)=1.235 p=0.2729	F(1,41)=1.281 p=0.2642
REM	F(1,41)=1.190 p=0.2817	F(1,41)=9.989 p=0.0030	F(1,41)=6.924 p=0.0119
Total length DG	F(1,8)=18.72 p=0.0025	F(1,8)=0.5896 p=0.4646	F(1,8)=0.9597 p=0.3559
N of Branches DG	F(1,8)=48.67 p=0.0001	F(1,8)=4.079 p=0.0781	F(1,8)=4.808 p=0.0597
Total length V1	F(1,12)=36.24 p<0.0001	F(1,12)=2.466 p=0.1423	F(1,12)=0.3797 p=0.5494
N of Branches V1	F(1,12)=57.08 p<0.0001	F(1,12)=4.547 p=0.0543	F(1,12)=0.0090 p=0.9262
Dendritic spine CA1	F(1,12)=24.39 p=0.0003	F(1,12)=0.2116 p=0.6538	F(1,12)=1.1170 p=0.3115
Dendritic spine V1	F(1,13)=67.58 p<0.0001	F(1,13)=1.107 p=0.3118	F(1,13)=0.0887 p=0.7705
$VGluT1^+Homer1bc^+ /VGluT1^+$ S1 Layer II-III	F(1,12)=50.26 p<0.0001	F(1,12)=26.59 p=0.0002	F(1,12)=1.2 p=0.29
$VGluT1^+Homer1bc^+ /VGluT1^+$ S1 Layer V	F(1,12)=43.57 p<0.0001	F(1,12)=2.36 p=0.15	F(1,12)=3.4 p=0.09
$VGluT1^+Homer1bc^+ /VGluT1^+$ V1 Layer II-III	F(1,12)=19.94 p=0.0008	F(1,12)=0.88 p=0.36	F(1,12)=2.33 p=0.15
$VGluT1^+Homer1bc^+ /VGluT1^+$ V1 Layer V	F(1,10)=31.47 p=0.0002	F(1,10)=29.87 p=0.0003	F(1,10)=0.06 p=0.80
c-Fos+ cells CA1	F(1,16)=14.11 p=0.017	F(1,16)=0.9043 p=0.356	F(1,16)=0.1460 p=0.707
c-Fos+ cells S1	F(1,21)=19.86 p=0.0002	F(1,21)=0.5041 p=0.485	F(1,21)=2.543 p=0.126
c-Fos+ cells V1	F(1,12)=36.14 p<0.0001	F(1,12)=0.5311 p=0.480	F(1,12)=0.0309 p=0.863
Hoechst+ cells CA1	F(1,12)=35.91 p<0.0001	F(1,12)=1.674 p=0.2200	F(1,12)=0.7887 p=0.3906
Pyknotic+ cells CA1	F(1,12)=1.790 p=0.2057	F(1,12)=3.482 p=0.0866	F(1,12)=1.963 p=0.1865
NeuN+ cells CA1	F(1,13)=126.6 p<0.0001	F(1,13)=70.65 p<0.0001	F(1,13)=1.534 p=0.2375
Cleaved caspase 3+ cells CA1	F(1,12)=6.750 p=0.0303	F(1,12)=6.030 p=0.0303	F(1,12)=1.770 p=0.2081
Cleaved caspase 3+cells S1	F(1,12)=4.213 p=0.0626	F(1,12)=7.659 p=0.0170	F(1,12)=2.836 p=0.1180
TH+ cells	F(1,14)=0.0118 p=0.9151	F(1,14)=10.06 p=0.0068	F(1,14)=9.169 p=0.0090
SA-β-gal CA3	F(1,10)=24.15 p=0.0006	F(1,10)=32.40 p=0.0002	F(1,10)=16.84 p=0.0021
SA-β-gal CA1	F(1,10)=16.64 p=0.0022	F(1,10)=4.647 p=0.0565	F(1,10)=7.405 p=0.0215
SA-β-gal S1	F(1,10)=15.84 p=0.0026	F(1,10)=4.806 p=0.0531	F(1,10)=2.000 p=0.1877
γH2AX CA3	F(1,9)=271.6 p<0.0001	F(1,9)=65.47p<0.0001	F(1,9)=0.0537 p=0.8220
γH2AX CA1	F(1,9)=70.41 p<0.0001	F(1,9)=30.85 p=0.0004	F(1,9)=0.0890 p=0.7722
Western Blot (XRCC5/GAPDH)	F(1,12)=25.50 p=0.0003	F(1,12)=13.01 p=0.0036	F(1,12)=1.133 p=0.3081
Western Blot (γH2AX /GAPDH)	F(1,11)=190.0 p<0.0001	F(1,11)=90.04 p<0.0001	F(1,11)=18.53 p=0.0012
Non-invasive transcranial intrinsic optical signal (IOS)	F(1,26)=22.60 p<0.0001	F(1,26)=0.7350 p=0.3991	F(1,11)=0.0877 p=0.7694
Percentage of spine CA1	F(1,12)=16.18 p=0.0017	F(1,12)=25.79 p=0.0003	F(1,12)=1.235 p=0.2883
Percentage of spine V1	F(1,13)=36.04 p<0.0001	F(1,13)=51.05 p<0.0001	F(1,13)=0.6339 p=0.4402
Homer1bc S1 puncta Layer II/III	F(1,13)=14.60 p=0.0019	F(1,13)=1.681 p=0.216	F(1,13)=0.5207 p=0.482
Homer1bc S1 puncta Layer V	F(1,13)=8.926 p=0.010	F(1,13)=0.8633 p=0.370	F(1,13)=0.0144 p=0.906
Homer1b V1 puncta Layer II/III	F(1,10)=15.05 p=0.0031	F(1,10)=2.113 p= 0.177	F(1,10)=0.6219 p=0.450
Homer1b V1 puncta Layer V	F(1,10)=21.41 p=0.0009	F(1,10)=0.995 p=0.342	F(1,10)=0.0249 p=0.880
GFAP+ cells CA1	F(1,12)=3.539 p=0.0844	F(1,12)=1.065 p=0.3224	F(1,12)=1.905 p=0.1927
S100+ cells V1	F(1, 12)=96.85 p<0.0001	F(1, 12)=1.791 p=0.2056	F(1, 12)=6.623 p=0.0244

© 2020. Gennaccaro L et al. Published online at http://www.aginganddisease.org/EN/10.14336/AD.2020.0827

SUPPLEMENTARY DATA

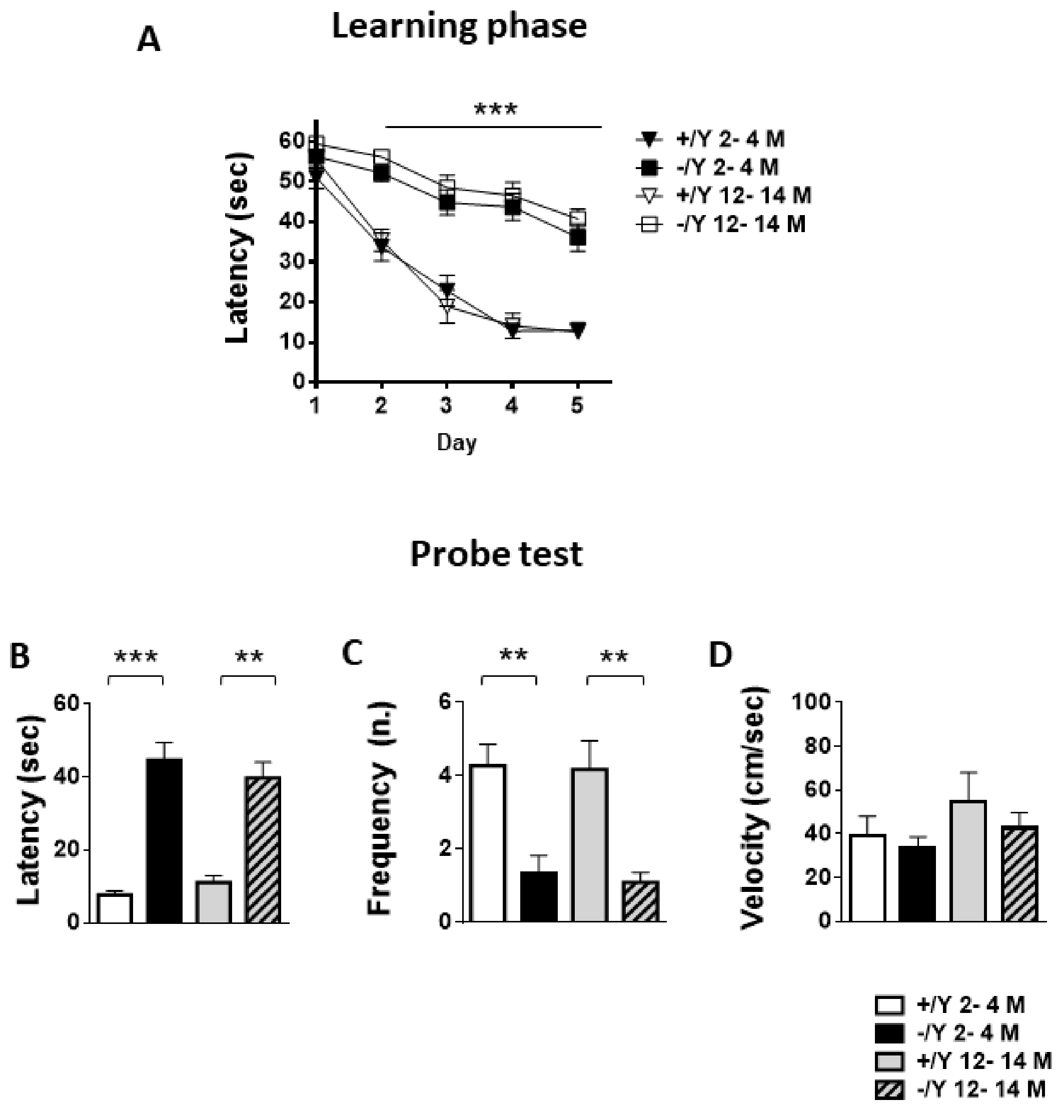

Supplementary Figure 1. Age-dependent effects on hippocampus-dependent learning and memory performance in *Cdkl5* KO mice. A) Spatial learning assessed with the Morris Water Maze in *Cdkl5* KO mice. Young adult *Cdkl5* -/Y (n = 17) and middle-aged *Cdkl5* −/Y (n = 21) mice showed an increased latency to find the platform over the 5-day learning period compared to their wild-type counterparts (young adult *Cdkl5* +/Y (n = 18) and middle-aged *Cdkl5* +/Y (n = 11) mice). **B-D)** On day 6 (probe test), the platform was removed and memory was assessed by evaluating the latency to enter the former platform zone (B), the frequency to enter in the quadrant in which the platform had been located during the learning period (C) and the maximum swim speed during the probe test (D) in mice as in (A). Values represent mean ± SE. ***p< 0.001 as compared to the *Cdkl5* +/Y matched for age. Dataset in (A), Fisher's LSD after three-way mixed ANOVA; datasets in (B-D), Fisher's LSD after two-way ANOVA.

© 2020. Gennaccaro L et al. Published online at http://www.aginganddisease.org/EN/10.14336/AD.2020.0827

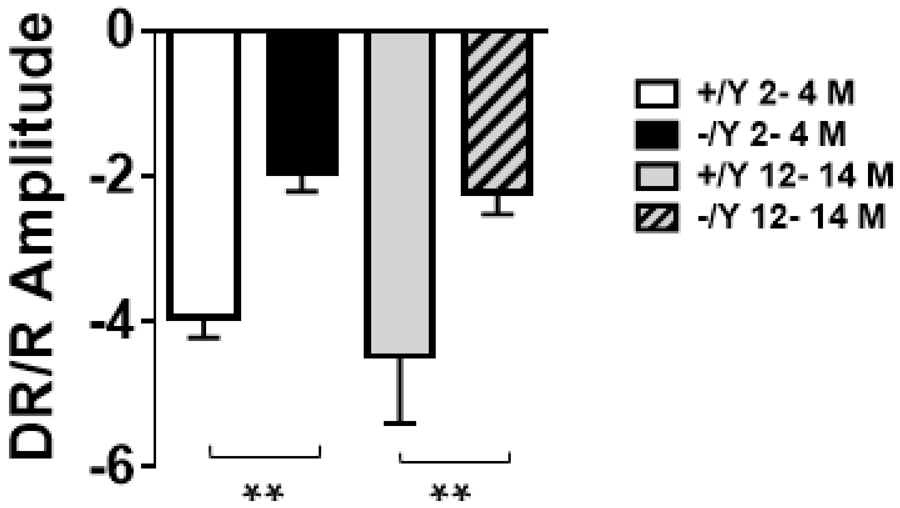

Supplementary Figure 2. Cortical visual responses are impaired in young adult and middle-aged *Cdkl5* KO mice. Quantitation of the average amplitude of the intrinsic signal between the experimental groups. Young adult *Cdkl5* -/Y (n = 6) and middle-aged *Cdkl5* −/Y (n = 7) mice showing a significant reduction of the evoked visual responses respect to both control groups (young adult *Cdkl5* +/Y (n = 9) and middle-aged *Cdkl5* +/Y (n = 8) mice). Values are represented as means ± SE. *p< 0.05 and **p< 0.01, ***p< 0.001 (Fisher's LSD after two-way ANOVA).

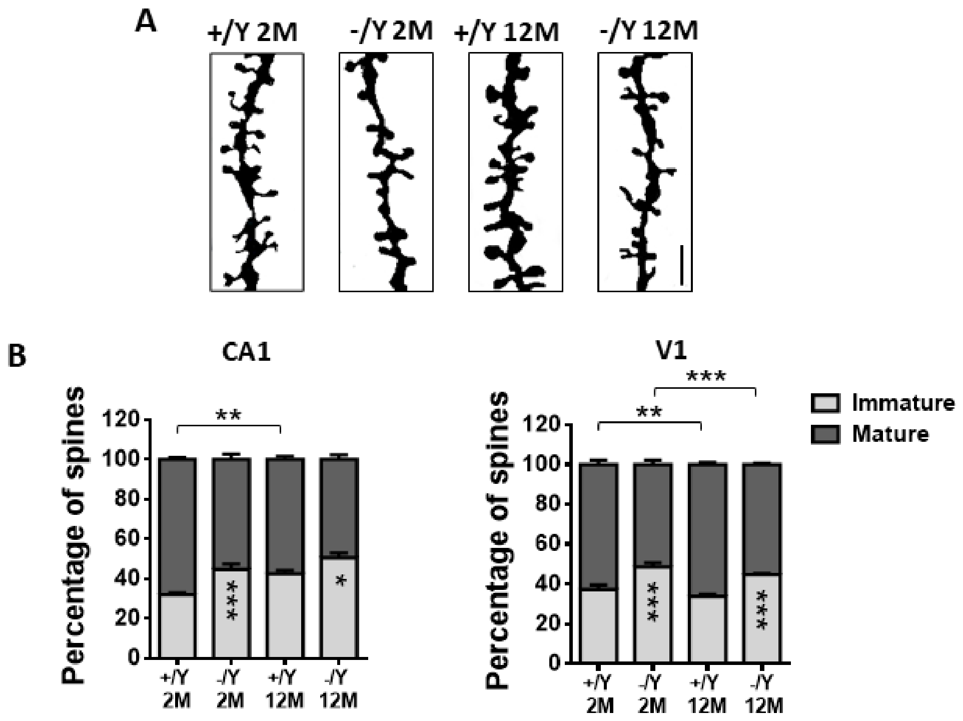

Supplementary Figure 3. Impaired hippocampal morphology in young adult and middle-aged *Cdkl5* KO mice. A) Examples of Golgi-stained dendritic branches of in CA1 pyramidal neurons of one animal from each experimental group. Scale bar = 1 µm. **B)** Percentage of immature and mature spines in relation to the total number of protrusions in CA1 pyramidal neurons and V1 pyramidal neurons of adult *Cdkl5* -/Y (n = 4) and *Cdkl5* +/Y (n = 4) mice, and middle-aged *Cdkl5* -/Y (n = 4) and *Cdkl5* +/Y (n = 4) mice. Values in B are represented as meas ± SE. *p< 0.05 and **p< 0.01, ***p< 0.001 (Fisher's LSD after two-way ANOVA).

© 2020. Gennaccaro L et al. Published online at http://www.aginganddisease.org/EN/10.14336/AD.2020.0827

SUPPLEMENTARY DATA

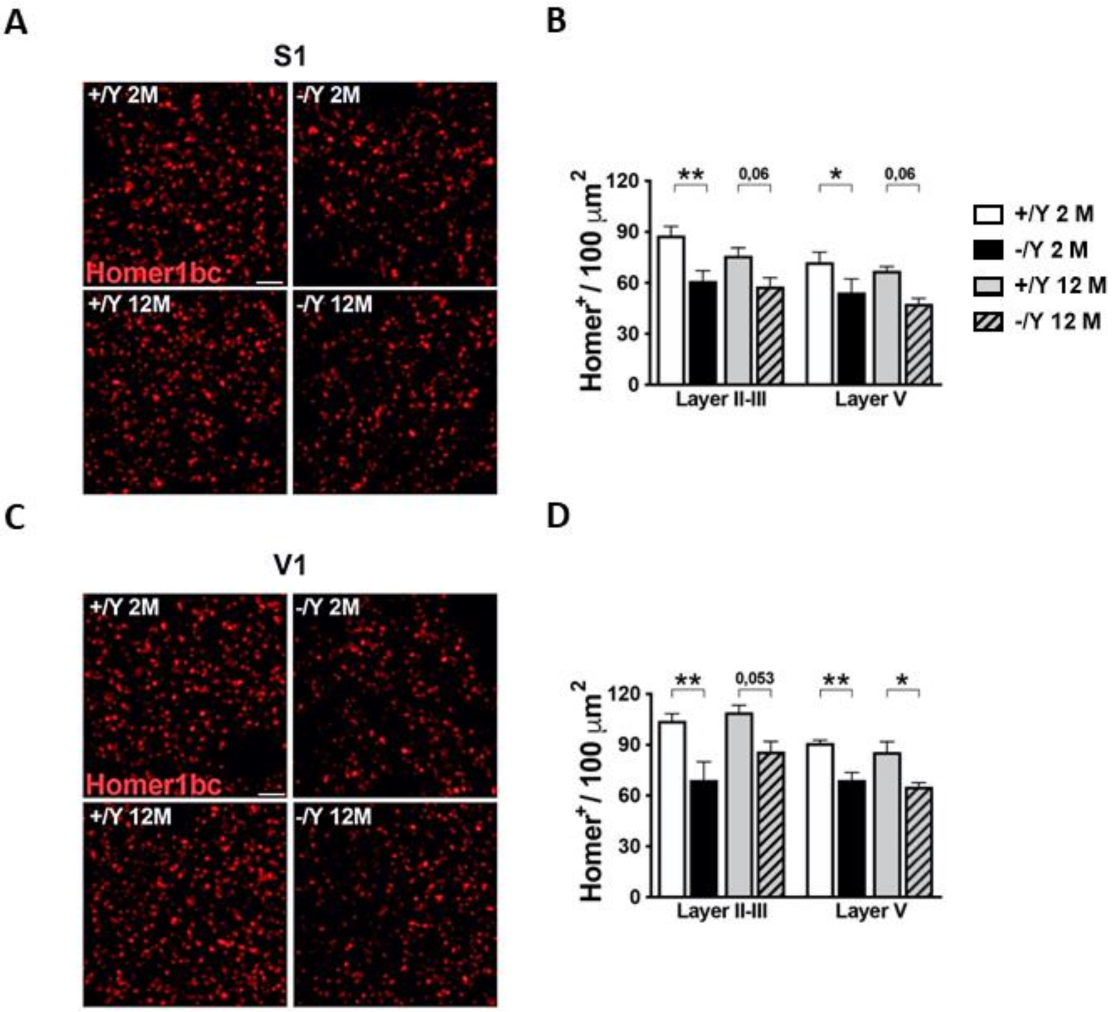

Supplementary Figure 4. Aberrant organization of the excitatory scaffolding protein Homer1bc in the cerebral cortex of young adult and middle-aged *Cdkl5* KO mice. A, C) Representative confocal micrographs of the neuropil (layer II/III) of S1 (A) and V1 (C) from young and middle-aged *Cdkl5* +/Y and *Cdkl5* -/Y mice showing the punctuate immunostaining of Homer1bc. Scale bar = 5 μm. **B)** Quantitative analysis of immunoreactive synaptic puncta of Homer1bc in the S1 cortex from young adult *Cdkl5* +/Y (n = 5) and *Cdkl5* -/Y mice (n = 5) and middle-aged *Cdkl5* +/Y (n = 4) and *Cdkl5* -/Y mice (n = 4). **D)** Quantification analysis of immunoreactive synaptic puncta of Homer1bc in the V1 cortex of young adult *Cdkl5* +/Y (n = 3) and *Cdkl5* -/Y mice (n = 4) and middle-aged *Cdkl5* +/Y (n = 4) and *Cdkl5* -/Y mice (n = 3). Values are represented as means ± SE. *p< 0.05, **p< 0.01, ***p< 0.001 (Fisher's LSD test after two-way ANOVA).

© 2020. Gennaccaro L et al. Published online at http://www.aginganddisease.org/EN/10.14336/AD.2020.0827

SUPPLEMENTARY DATA

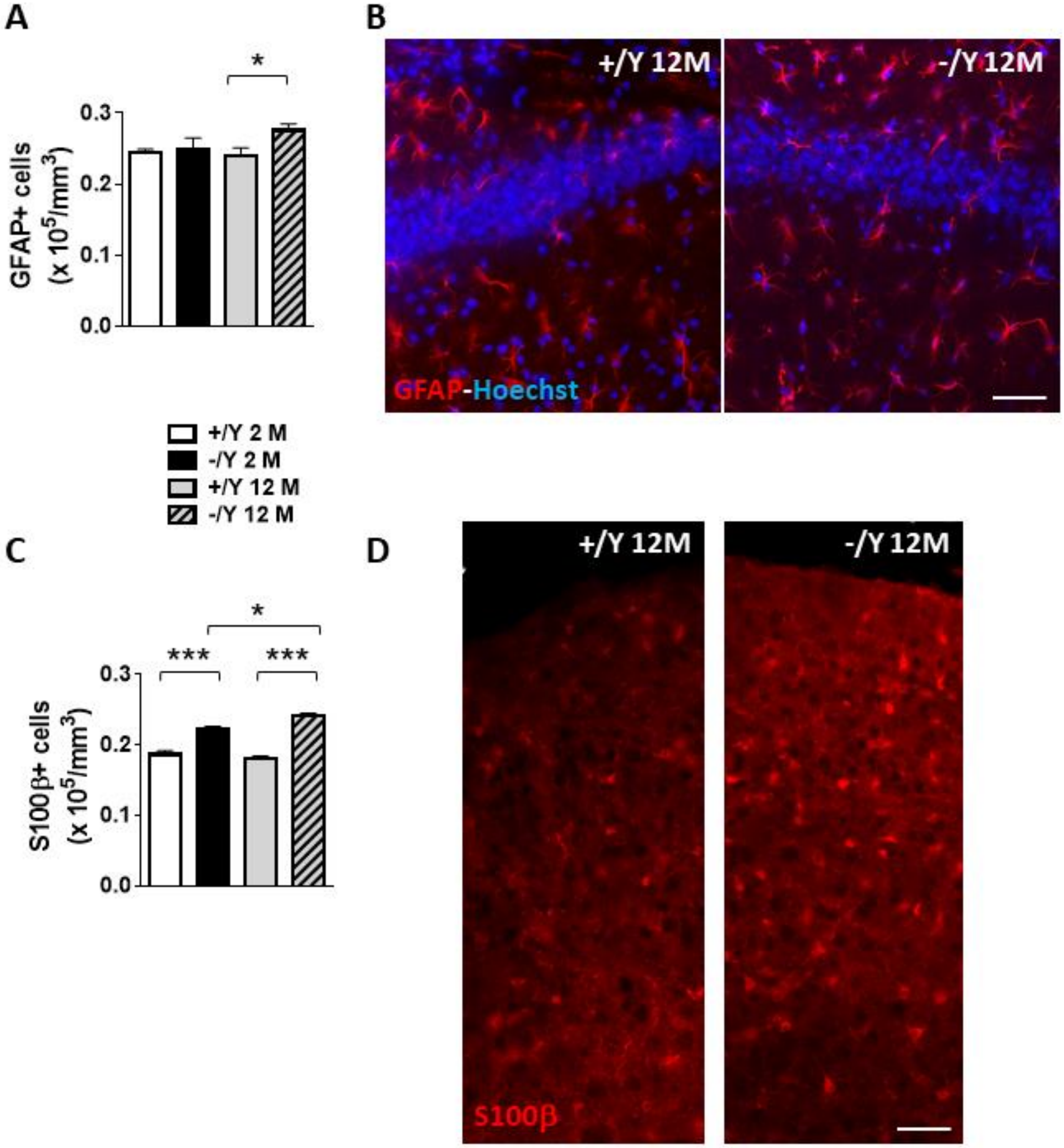

Supplementary Figure 5. Hippocampal GFAP-immunoreactive cells were examined in young adult and middle-aged *Cdkl5* KO mice by immunofluorescence labeling. A) Quantification of GFAP immunoreactive cells in the hippocampal CA1 region from young adult *Cdkl5* +/Y (n = 4) and *Cdkl5* -/Y (n = 4) mice, and middle-aged *Cdkl5* +/Y (n = 4) and *Cdkl5* -/Y (n = 5) mice. **B)** Representative fluorescent images of hippocampal CA1 pyramidal cell layer of middle-aged *Cdkl5* +/Y and a *Cdkl5* -/Y mouse immunostained for GFAP and counterstained with Hoechst. Scale bar = 50 µm. **C)** Quantification of S100β-immunoreactive cells in the primary visual cortices V1 from young adult *Cdkl5* +/Y (n = 4) and *Cdkl5* -/Y (n = 4) mice, and middle-aged *Cdkl5* +/Y (n = 4) and *Cdkl5* -/Y (n = 5) mice. **D)** Representative fluorescent images from the pyramidal cell layer of the primary visual cortices V1 of middle-aged *Cdkl5* +/Y and a *Cdkl5* -/Y mouse immunostained for S100β. Scale bar = 50 µm. Values are represented means ± SE. *p<0.05, **p<0.01, ***p<0.001 (Fisher's LSD test after two-way ANOVA).

© 2020. Gennaccaro L et al. Published online at http://www.aginganddisease.org/EN/10.14336/AD.2020.0827
